# The Role of Number of Copies, Structure, Behavior and Copy Number Variations (CNV) of the Y Chromosome in Male Infertility

**DOI:** 10.3390/genes11010040

**Published:** 2019-12-29

**Authors:** Fabrizio Signore, Caterina Gulìa, Raffaella Votino, Vincenzo De Leo, Simona Zaami, Lorenza Putignani, Silvia Gigli, Edoardo Santini, Luca Bertacca, Alessandro Porrello, Roberto Piergentili

**Affiliations:** 1 Department of Obstetrics and Gynecology, Misericordia Hospital, 58100 Grosseto, Italy; fabrizio.signore@uslsudest.toscana.it (F.S.); raffaella.votino@uslsudest.toscana.it (R.V.); 2 Department of Urology, Misericordia Hospital, 58100 Grosseto, Italy; 85cate@live.it (C.G.); urologia.santini@gmail.com (E.S.); 3 Department of Molecular Medicine and Development, Università degli Studi di Siena, 53100 Siena, Italy; vincenzo.deleo@unisi.it; 4 Department of Anatomical, Histological, Forensic and Orthopedic Sciences, Sapienza University of Rome, 00161 Rome, Italy; simona.zaami@uniroma1.it; 5 Unit of Parasitology and Unit of Human Microbiome, IRCCS Bambino Gesù Children’s Hospital, 00165 Rome, Italy; lorenza.putignani@opbg.net; 6 Department of Diagnostic Imaging, Sandro Pertini Hospital, 00157 Rome, Italy; adrenalina_1@hotmail.it; 7 Pediatric Emergency Unit, Misericordia Hospital, 58100 Grosseto, Italy; darra80@hotmail.com; 8 Lineberger Comprehensive Cancer Center, University of North Carolina at Chapel Hill, Chapel Hill, NC 27599, USA; 9 Institute of Molecular Biology and Pathology of the Italian National Research Council (IBPM-CNR), Sapienza University of Rome, 00185 Rome, Italy

**Keywords:** Copy number variations (CNV), karyotype, mosaicism, epigenetics, aneuploidy

## Abstract

The World Health Organization (WHO) defines infertility as the inability of a sexually active, non-contracepting couple to achieve spontaneous pregnancy within one year. Statistics show that the two sexes are equally at risk. Several causes may be responsible for male infertility; however, in 30–40% of cases a diagnosis of idiopathic male infertility is made in men with normal urogenital anatomy, no history of familial fertility-related diseases and a normal panel of values as for endocrine, genetic and biochemical markers. Idiopathic male infertility may be the result of gene/environment interactions, genetic and epigenetic abnormalities. Numerical and structural anomalies of the Y chromosome represent a minor yet significant proportion and are the topic discussed in this review. We searched the PubMed database and major search engines for reports about Y-linked male infertility. We present cases of Y-linked male infertility in terms of (i) anomalies of the Y chromosome structure/number; (ii) Y chromosome misbehavior in a normal genetic background; (iii) Y chromosome copy number variations (CNVs). We discuss possible explanations of male infertility caused by mutations, lower or higher number of copies of otherwise wild type, Y-linked sequences. Despite Y chromosome structural anomalies are not a major cause of male infertility, in case of negative results and of normal DNA sequencing of the ascertained genes causing infertility and mapping on this chromosome, we recommend an analysis of the karyotype integrity in all cases of idiopathic fertility impairment, with an emphasis on the structure and number of this chromosome.

## 1. Introduction

The World Health Organization (WHO) defines infertility as the inability of a sexually active, non-contracepting couple to achieve spontaneous pregnancy within one year [1]. Many studies focused on the possible causes of infertility, and it has been proven that, globally, both sexes are equally involved [2]. Traditionally, male infertility has been divided into three major etiological categories: Pretesticular, testicular, and post-testicular [3]; however, another new clinically based classification has been recently proposed, considering: (a) The hypothalamic–pituitary axis function, (b) quantitative spermatogenesis, (c) qualitative spermatogenesis, and (d) ductal obstruction or dysfunction [4], all of these including both genetic and non-genetic causes. Focusing also on the clinical characteristics of the patient and not only on the anatomical/temporal characteristics of infertility might be a better approach in the diagnosis and treatment of this condition, since male infertility can be caused by several, different factors that may affect semen quality in very different ways, so that this quality can be seen as the final output of different pathophysiological mechanisms. These two classification methods are summarized in Table 1 to highlight similarities and differences better. Male fertility can be influenced by congenital or acquired urogenital anomalies, malignancies, urological infections, varicocele, genetic abnormalities, endocrine disorders and immunological factors [5]. However, it is not uncommon to diagnose idiopathic male infertility in men with normal anatomy, no history of familial fertility-related diseases and a normal panel of values as for endocrine, genetic and biochemical markers: In fact, in 30–40% of cases no male infertility-associated factor is found [5]. Azoospermia is the absence of spermatozoa from the seminal fluid and can be caused by non-obstructive (80–85%) or obstructive events (15–20%) although these values may significantly vary according to different studies [6]. Obstructive azoospermia can be further divided into (a) intratesticular (post-inflammatory or post-traumatic), (b) epidydimal (secondary to epididymitis or chronic infections), (c) vas deferens (post-vasectomy), (d) ejaculatory duct (cystic or post-inflammatory), and (e) distal seminal ducts (local neuropathy) obstruction [7]. Finally, environmental factors, such as tobacco smoking and assumption of steroids, seemingly also play a role in altering male fertility [8]. Prognostic factors for male infertility involve the analysis of semen quality, which includes both sperm quantity and quality. Table 2 reports the minimum values of semen features, according to the WHO data, for normozoospermic men [9]. Recent studies demonstrate a general decline of semen quality in Western countries [10] without a clearly identified cause. Age is another cause of decreased semen quality, which affects the genetic quality of sperm (damaged DNA), its volume, spermatozoa motility and morphology [11]. Thus, in the standard laboratory tests (Table 2), semen quality is defined as follows: Oligozoospermia if there are <15 million spermatozoa/ml; asthenozoospermia if there are <32% of progressively motile spermatozoa; teratozoospermia if there are <4% of normal forms. It is also important to evaluate if infertility is primary or secondary, i.e., if the couple has become pregnant at least once in the past; this helps to discriminate congenital from acquired causes.

In this review, we discuss the male sterility caused by alterations in the shape, overall content or behavior of the Y chromosome, and describe how the size variation of specific regions of this chromosome or Y chromosome aneuploidies, irrespectively of point mutations in coding gene sequences, affects the male fertility.

## 2. Overview of the Y Chromosome Evolution and Structure

**Figure 1:**
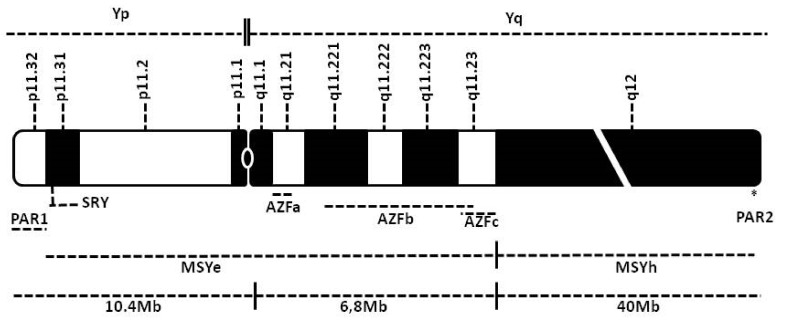
Overview of the Y chromosome organization. Rows from the top: (1) Short (Yp) and long (Yq) arms of the Y chromosome; (2) numbered cytogenetic bands; (3) chromosome banding according to the US National Library of Medicine, retrieved on October 25, 2018, at https://ghr.nlm.nih.gov/chromosome/Y#idiogram (note: The region q12 is shortened (white cut), due to its large extension compared to the rest of the chromosome); (4) localization of the *SRY* gene, of the PAR regions (PAR2 is indicated by an asterisk, due to its small size) and of the AZF regions; (5) extension of the euchromatic (MSYe) and heterochromatic (MSYh) portions of the MSY region; (6) approximate length in megabases (millions of base pairs, Mb) of the short arm, euchromatic portion of the long arm and heterocromatic portion of the long arm.

The two most important features of the genetic behavior of human Y-chromosomes are the lack of homologous chromosome exchanges for many of its regions (except for PAR1 and PAR2, as described below), and a male-limited transmission of the male-specific Y (MSY) segment [13] (Figure 1). Sex chromosomes are derived from autosomes [14,15,16], and encode the master-sex determining proteins, i.e., Testis Determining Factor (TDF) on the Y (see the section ‘The Sex-Determining Region of the Y (SRY)’) and Androgen Receptor (AR) on the X [17]. Massive gene decay and transposition have characterized the Y chromosome evolution [18,19], which only contains 3% of the genes of its ancestor [20]; for comparison, the Y chromosome harbors 54 protein-coding genes, while the X chromosome hosts ~700 [21]. Consequently, compared to the other human chromosomes, the Y chromosome has a limited number of genes, since they tended to degenerate during the evolution [22,23], showing a high degree of variability, among which copy number variants contribute the greatest predicted functional impact [24,25]. Overall, the Y chromosome is considered the relic of ancient autosomes that became the ancestors of both allosomes through sequential chromosomal fusions and rearrangements [20]. This karyotipe evolution paralleled that of the genetic sequences mapping on both sex chromosomes, which indeed are enriched in dosage-sensitive genes, mirroring the gene dosage compensation mechanism typical of mammalian sex chromosome biology [20,26]. In particular, it has been shown that the genes in common between the sex chromosomes are enriched for important regulatory functions and predictors of haploinsufficiency [20,27,28]. The Y chromosome (comprising over 57 million base pairs); contains genes and highly repetitive DNA sequences, which harbor pseudogenes without any known function [29].

## 3. Mosaics and Y Chromosome Structural Anomalies

### 3.1. Mosaics

Because of a mis-segregation of the sex chromosomes during the very early stages of development, some individuals may become mosaics, i.e., in the simplest case they harbor a mixture of cells whose karyotype is either 46,XY or 45,X [30]. The status of these patients’ gonads is largely unpredictable, and deeply influences their phenotype and sexual habitus. The most extreme cases are bilateral nonfunctional rudimentary streak gonads (female habitus; typical symptom: Missing puberty) and bilateral small scrotal testes (male habitus; typical symptom: Infertility consequent to azoospermia or severe oligospermia). All possible intermediate situations have been described. As a consequence, the patient’s habitus may be male, female or ambiguous, depending on the extension, localization and time of formation of mosaic patches, or based on other, rarer mechanisms, such as chimerism [31]. The gonadal function is, in turn, influenced by the number of cells that either have or do not a Y chromosome; indeed, there seems to be a minimum threshold of Y-bearing cells in the gonadal ridge that allows the development of a male habitus [32]. Notably, the percentage of SRY-positive cells in the gonads may be significantly different from that of the peripheral blood cells (the standard tissue used for karyotyping) because the embryological origin of the latter cells is in the mesoderm, while the former cells arise extragonadally in connection with the endoderm [33]. A similar situation is described for buccal cells of ectodermic origin [32]. Relying on the formation of mosaics in patients with rearranged, dicentric Y chromosomes, some authors suggest that a greater instability of the aberrant Y chromosome might promote its loss during the cell divisions of the germline, thus, promoting the development of a female habitus [34].

Among all mosaic patients, those with a male phenotype are characterized by bilaterally descended testes and represent 11–12% of the total [35,36,37]. The diagnosis of male mosaic patients usually occurs during fertility evaluations, upon discovery of azoospermia and elevated serum gonadotropins levels, consistent with testicular function failure [30]. Because of the increased risk of gonadoblastoma formation, present medical guidelines suggest removing the non-functional gonads, especially if this condition is diagnosed during childhood.

We conclude this section by recalling a recent work showing the connection between Y chromosome mis-segregation and its subsequent rearrangement(s) [38]. This study demonstrates that, in some cases (and if not simply lost), the mis-segregating Y chromosome, in the time frame of three consecutive cell cycles, is first included in a micronucleus, then fragmented, and finally, its fragments are re-joined through the error-prone Non-Homologous End Joining (NHEJ) mechanism (similarly to its autosomic counterparts [39]). This outcome sets the molecular and cytological bases for-at least some-of the chromosome aberrations described below.

### 3.2. Chromosome Rings and Isodicentric Chromosomes

Approximately half of the mosaic patients have an abnormal Y chromosome, shaped like a ring. Ring chromosomes of the Y (rY) have no free extremities (telomeres) and are shaped as circular DNAs. This rearrangement may be due to several causes, including telomeric fusions or terminal deletions of both arms that are subsequently (erroneously) attached together by DNA repair mechanisms. In the latter case, usually, rY-bearing cells do not have the Y-derived, terminal, acentric fragments, which are lost during cell cycles; the length of these lost fragments is extremely variable. rY are intrinsically unstable in the genome and prone to mis-segregation during cell division, mainly because of the sister chromatids entanglement during the replication phase, causing anaphase lags and chromosome breakage, leading to further rearrangements. In these cases, the mosaic usually is a consequence of a previously formed rY. These patients’ cells either have a numerically normal (46,X,r(Y)) or aneuploid (45,X; i.e., the Y chromosome is missing) karyotype. In a minority of these patients’ cells, there is the complementary situation, i.e., they have two rY chromosomes (47,X,2r(Y)), but this happens sporadically, because these cells also have increased genome instability. In other cells, the Y chromosome is further rearranged to create a single ring from two or more Y chromosomes, harboring a variable number of centromeric sequences [40]; this situation is sporadic and very unstable as well. In addition, interstitial deletions inside the rY are relatively frequent, even in distinct cells from the same patient [30]. All these conditions usually emerge de novo after fertilization or during the father’s meiosis, because of the sterility of patients with rY. The only exception, to the best of our knowledge, was described by Arnedo and collaborators in 2005 [41]. As a general rule, mosaic patients with a male habitus manifest gonadal failure and short stature, as well as increased prevalence of cardiorenal malformations and germ cell tumors [35,37]; otherwise, especially in the absence of additional, very long interstitial deletions, mosaic subjects are normal males, and their phenotypes are mainly related to the extension of Y-linked terminal lost sequences and to the total amount of aberrant cells.

Besides the further rearrangements of the ring chromosomes, Y chromosomes showing two or more centromeric sequences (isodicentric Y chromosomes, idicY) are also found in the absence of rY. Depending on the Y portion that is present, idicY can be associated with either a male or female phenotype [42,43]. These aberrant chromosomes arise, in most cases, as an output of homologous crossing-over between opposing arms of palindromes on sister chromatids [43,44]. These ectopic recombination events occur at nearly all Y-linked palindromes, excluding the smaller one (P7), although this might be just a consequence of the relatively small number of patients analyzed. In a minority yet a significant proportion of patients, idicY formation follows the recombination events occurring within the pericentromeric heterochromatin [43,44]. idicY are found at relatively high frequency in men with non-obstructive azoospermia, and this strong phenotype is likely due to the loss of important genes, mapping on the Y chromosome, consequent to the rearrangement. Interestingly, mosaicism (45,X-46,X,idicY) in these individuals is high [45]; in addition, there is the chance that one of the two centromeres in the Y chromosome is functionally inactive, in a direct relation with their distance (i.e., the longer the distance, the higher the probability of inactivation) [44]. Because of the intrinsic genomic instability of dicentric chromosomes, it is likely that both phenomena (loss of the aberrant Y chromosome and inactivation of the second centromere) occur because idicY-bearing cells are less viable and more unstable than the aneuploid ones.

### 3.3. Chromosome Translocations

Male patients may also have a 46,XX karyotype; they are identified as 46,XX testicular Disorder of Sex Development (DSD) [46], a.k.a. de la Chapelle syndrome [47]. The incidence of this condition is estimated to be 1/20,000 to 25,000 male births [48,49], and, to date, only a few hundred patients have been described [50]. Most of them show rearranged chromosomes, with a variable portion of the Y chromosome short arm—bearing the *SRY* gene—translocated to another chromosome. The most frequent occurrence is an X-Y translocation, consequent to an aberrant crossing-over during the father’s meiosis [51]. However, also autosome-Y (A-Y) translocations have been reported. If these translocations are not genetically balanced (the far more frequent event), several phenotypes are present, due to the missing loci from both the Y and the autosome involved. Instead, in a few cases, it has been shown that a balanced A-Y translocation without significant DNA loss might be transmitted across several generations without impairing the male fertility [52]. About 80% of the translocated X-Y individuals have normal pubic hair growth after puberty and normal penis sizes, but usually also have small testicles, and their infertility is caused by azoospermia, consequent to AZF loci loss. Gynecomastia is present in about 30% of cases [53]. The male habitus of these individuals is typically attributed to the presence of the *SRY* gene, which acts as a dominant determinant of sex development; indeed, the *SRY* gene function is positive in approximately 80% of these individuals [53]. However, the mere presence of *SRY* is not sufficient for masculinization, as X inactivation in XX males may play an important role in *SRY*-positive XX males. This occurs because the *SRY*-bearing X chromosome is activated during the development in 90% of cases, while in the remaining cases *SRY* is inactivated together with the X chromosome it is attached to, thus, causing masculinization failure [54]. *SRY*-negative, infertile men have been reported as well [55,56,57]. In these patients, the virilization is not directly due to the Y chromosome behavior, but to a mutation that either causes the over-expression of X-linked or autosomal genes promoting masculinization (such as *SOX3*, *SOX9*, *SOX10*) or the down-regulation of autosomal genes blocking it (such as *WNT4* and *RSPO1*) [58,59]. On the X chromosome, a similar habitus is caused by loss-of-function mutations in the Androgen Receptor (AR) coding gene [17,50]. Since masculinization, in these cases, is not strictly driven by the Y chromosome, we will not discuss them.

## 4. Y Chromosome Aneuploidies

### 4.1. Disomy of the Y

Y chromosome hyperploidies are important for male infertility, since they can unveil the possible role of additional—but otherwise, wild type–copies of genes mapping on Y. Differently from what described in the previous section, in this case, all patients’ cells have the same karyotype since the mis-segregation occurs before fecundation, i.e., in the father’s germ line. During meiosis, homologous chromosomes segregate in secondary spermatocytes and then sister chromatids further segregate in spermatids. Meiosis impairment may cause chromosome mis-segregation and, if sex chromosomes are involved, the resulting gametes will produce, upon fecundation, aneuploid individuals. Most commonly, the offspring will be affected either by Turner (45,X karyotype, female habitus) or by Klinefelter syndrome (47,XXY karyotype, male habitus). Both syndromes are considered a consequence of the meiotic behavior of the X chromosome [60] either in the father or in the mother; thus, they are outside the scope of this review.

Sporadically, during the father’s meiosis II, the two sister chromatids of the Y chromosome do not segregate, causing the formation of an aberrant sperm having 24 chromosomes and containing two Ys. If this sperm fertilizes a normal egg, the individual will have a 47,XYY karyotype (a condition known as XYY syndrome or Jacobs syndrome); thus, the XYY syndrome is truly related to Y chromosome aneuploidy [61]. This happens in about 1 in 1,000 newborn boys [62], and is the most common sex chromosome aneuploidy in males after the Klinefelter syndrome. The exact causes of this mis-segregation are unknown, but the meiotic recombination between X and Y may play a role [63], as well as other, unidentified causes that affect multiple chromosome segregations at the same time [64]. Indeed, the meiotic recombination-dependent mis-segregation of X and Y chromosomes is a known cause of male infertility in otherwise karyotipycally normal men [65]. More generally, sperm aneuploidy of the sex chromosomes affects fertile men as well, though without impairing their ability to procreate [66]. The phenotype of 47,XYY subjects is generally almost normal, except for a slightly taller stature, variable cognitive disorders and, more rarely, motor skill impairment [60,67,68]. The fertility of these individuals is controversial. Some reports showed that often there is no significant difference between XYY males and control groups with regard to the number of children, and the children of XYY males were as healthy as those of the control group [69,70]. This explains why usually this condition is discovered only in adults. Other researchers, instead, report fertility impairment, generally due to an increased incidence of chromosomally abnormal spermatozoa; these men have variable sperm counts, ranging from almost-normospermia to oligoasthenoteratozoospermia, to azoospermia [61,71] (and references therein). In some cases, secondary infertility has been described [61]. Cases of infertility of these individuals are explained, by some authors, as a consequence of the specific loss of one copy of the Y chromosome before meiosis or of specific degeneration of aneuploid gametes through apoptosis or necrosis [72,73,74,75]. Other authors, instead, showed that a couple of Y chromosomes could synapse in meiosis, thus, escaping the meiotic sex chromosome inactivation which, in turn, would keep active genes that should not be such, hence, inducing fertility impairment [76]. Finally, the karyotype of some patients is a mosaic of 46,XY and 47,XYY cells. They may have very mild or no fertility issues, mainly depending on the count of 47,XYY cells in their gonads; if the 47,XYY count is very low, these patients might not be classified as mosaics at all [61].

### 4.2. Sex Chromosomes Multisomy

More complex situations have been described, with males having tetrasomy or pentasomy of sex chromosomes and carrying multiple copies of the Y chromosome [77]. The 48,XXYY syndrome is generally considered the most common variant of the Klinefelter syndrome (47,XXY); however, it has been suggested that this condition is different [78,79]. The incidence of this condition is estimated between 1:50,000 [80], and 1:18,000 [81] male births. The associated phenotypes mostly resemble those of patients with 47,XXY karyotype [77]. Similarly to 47,XXY patients, some 48,XXYY subjects may be unaware of their condition, which, sometimes, is diagnosed at a later age [77]. These men typically show hypergonadotropic hypogonadism (as in Klinefelter individuals) with increased follicle-stimulating and luteinizing hormones, decreased testosterone and small testes, [77,82]. The infertility of these men is explained by the state of their gonads. Their testes show hyalinization of the seminiferous tubules, hyperplasia and fibrosis of interstitial (Leydig) cells, Sertoli-cell-only syndrome with lack of spermatogenesis [77,83,84]. The primary event for the generation of such individuals is a double sex chromosome mis-segregation in their fathers’ meiosis I and II or a contemporary mis-segregation of sex chromosomes in both parents [84]; a double sex chromosome mis-segregation in very early stages of embryonic development is another possible explanation.

Other, more complex conditions exist, but are extremely rare. However, since the phenotype of these patients depends on the polysomy of both sex chromosomes, the specific involvement of the Y chromosome is unclear. In this context, patients with a polysomy of the Y chromosome alone are more informative. In 48,XYYY patients, sometimes the diagnosis is made as adults; the oldest diagnosed case that we are aware of was a 52 years old subject affected by infertility and other, minor problems [85], indicating that this condition may be considered *mild*, at least in some circumstances. Indeed, the phenotype associated with an XYYY karyotype is inconsistent; genitalia are apparently normal, but show hypogonadism with azoospermia in most adult patients [77].

We were able to find only seven cases of 49,XYYYY patients without mosaicism, two of whom were adults. In an older report [86] the affected man (30 years old) showed azoospermia, elevated basal gonadotropins and an exaggerated testosterone rise following HCG stimulation. A newer report describes a 26 years old man originally diagnosed at the age of 4 months [87]; he was at first classified as a non-mosaic, but subsequent analyses showed that the 49,XYYYY karyotype was present in ca. 86–87% of his cells, the other cells being 45,X [88]. Genitalia were normally developed [87], and the gonadal function was normal as well [88], but, unfortunately, no additional data are available about his physiologic and spermatogenesis status. Nonetheless, data from mosaic patients suggest that the spermatogenesis of 49,XYYYY individuals is strongly impaired [89].

The paucity of data on patients having Y chromosome polysomy does not allow fully understanding the role of this chromosome on male infertility or in other phenotypes associated with supernumerary Ys; however, these patients are generally in better physical conditions than those with supernumerary Xs.

## 5. The PAR Regions

The human Y chromosome hosts two pseudoautosomal regions (PARs), named PAR1 and PAR2, which consist of 5% of the entire chromosome [90], and an MSY region (also known as non-recombining region on Y, NRY) that does not recombine with the X chromosome [91] (Figure 1). PAR1 and PAR2 recombine during meiosis with their homologous regions on the X chromosome, although with different dynamics, compared with the rest of the genome [92]. Indeed, genes located within the PARs are inherited, and genetically behave similarly to autosomal genes. Taken together, the two PARs host at least 29 genes [14], 24 in PAR1 and 5 in PAR2. PAR1 is 2.6 Mb long and maps on the short arm tips of both X and Y chromosomes in humans and other great apes; PAR1 escapes X inactivation, and mutations in its genes are known to cause short stature [93], growth retardation [94], and mental disorders [95,96]. PAR2 is located at the tips of the long arms and is much shorter, spanning only 320 kb [97]. The role of PAR1 in the recombination is crucial for male fertility. In mice, it has been demonstrated that recombination impairment leads to X-Y mis-segregation in meiosis [92]. Similarly, in humans, a fertility impairment has been described, which is caused by PAR1 deletions [98,99], and, likely, is due to a comparable X-Y meiotic mis-segregation [100,101,102]. Instead, PAR2 is not necessary to fertility, as shown in individuals bearing deletions of the Y chromosome [97]. This feature of PAR2 can be explained by assuming that (a) there are no genes inside it that are directly involved in male fertility, and (b) the recombination role of PAR1 is sufficient to assure the correct segregation of the sex chromosomes. In a minority (2%) of the human population, also a PAR3 has been described [103], but its functional role has not been elucidated yet. However, because of its low prevalence, PAR3 is unlikely to harbor sequences important for chromosome segregation or genes having any role in male fertility.

Some genes mapping in the PARs are expressed in testes, but they are also always expressed in other tissues, and their exact role in the spermatogenesis—if any—is currently unknown [104]. These genes map both in PAR1 (16 genes) and PAR2 (2 genes), but the limited data available about phenotype alterations based on their copy number variations (CNV) and their involvement in male fertility is still unclear. Here we only recall the debate regarding the *SHOX* gene, mapping in PAR1. In 2011, Jorgez and collaborators [105] reported the occurrence of *SHOX* gene deletion in 5.4% of men with AZF deletions and a normal karyotype, hypothesizing a correlation between PAR rearrangements and AZF microdeletion formation. However, subsequent studies did not confirm this association between Y-chromosomal microdeletions and *SHOX* haploinsufficiency [106,107].

## 6. The Sex-Determining Region of the Y (SRY)

The Y chromosome promotes sexual differentiation in humans. In fact, its presence (or absence) is strictly linked to the development of primary and secondary sexual features, due to the activity of the *Sex-determining Region Y* (*SRY*) gene, which comprises a 35-kb region of Y-specific DNA adjacent to the pseudoautosomal boundary [108] (Figure 1). This intronless sex-determining gene encodes a transcription factor, known as Testis-Determining Factor (TDF), which is a member of the high mobility group (HMG)-box family of DNA-binding proteins and is responsible for the initiation of male sex determination during embryonic life. This protein switches the undifferentiated genital ridge towards testis development: The absence of the SRY gene or the functional impairment of the TDF protein leads to the development of a female habitus, irrespective of the karyotype. This developmental switch is promoted by alternative genetic cascades, including female sex-determining genes *R-Spondin 1* (*RSPO1)*, *Wnt Family Member 4* (*Wnt4)/β-catenin* and *Forkhead Box L2* (*Foxl2)* [109]. The major role of *SRY* is to achieve, through enhancer sequence binding*,* a sufficient expression of the gene *Sox9*, in order to induce the Sertoli cell differentiation, which in turn leads to testis formation and prevents the development of female reproductive structures (uterus and fallopian tubes) [110]. Deletions or translocations of *SRY* cause disorders of sex development (DSD) with dysgenic gonads in mutated individuals: Affected patients may have external genitalia that are not clearly male or female (ambiguous genitalia) or other abnormalities of the genitals and reproductive organs, either in karyotypically male (XY) individuals (in case of *SRY* absence/total impairment) or in karyotypically female (XX) patients (in case of *SRY* translocation on the X chromosome or on the autosomes). Failures in the gonad development may result not only in infertility, but also an increased risk of germ cell tumours (GCT), such as gonadoblastoma and various types of testicular GCT [111,112]. Indeed, recent studies demonstrate that either the loss of the Y chromosome or the ectopic expression of Y-linked genes is closely associated with various male-biased diseases, including selected somatic cancers [113]. Amongst the most important disorders caused by mutations of the *SRY* gene we mention here the Swyer Syndrome (XY karyotype, female habitus with gonadal dysgenesis) and the XX male syndrome, in which part of the Y chromosome is translocated on the X chromosome as the result of unequal recombination in the distal parts of the short arms of the sex chromosomes during the paternal meiosis, determining *SRY*-positivity [114]. Nonetheless, in some cases with complex chromosome rearrangements or mosaicism, the mere presence of *SRY* seems insufficient to determine the full development of male genitalia [115,116]. It has been shown that, at least in some cases, two copies of wild type *SRY* do not affect male fertility and that tandem duplication of *SRY* and Y chromosome aneuploidy are independent events [117]. Unfortunately, the same report does not clarify the possible role of multiple *SRY* copies, since in patients with 4 to 16 copies of the gene, its DNA sequence was not wild type and/or X0/XY mosaicism was present, sometimes resulting in a female habitus (see also the section ‘Mosaics and Y structural anomalies’) [117]. In conclusion, due to its dominant action, CNV of *SRY* affect the phenotype only when *SRY* is missing from the Y chromosome or when it is translocated onto another chromosome; instead, to the best of our knowledge, no phenotypic effect is induced by additional, wild type copies of *SRY* alone (tandem duplications). DSD in the presence of multiple copies of *SRY* were either associated with point mutations, and other gene sequence alterations [117] or a clear cause-effect relationship between the former and the latter was not demonstrated [118]. As such, *SRY* is not a good candidate gene for searching causes of male infertility; its mutations (gene deletions or point mutations, mostly inside the HMG region) usually induce a female—or, at most, an ambiguous—phenotype. Instead, in the case of a male phenotype, the most frequent cause is the presence of mosaicism beyond a certain threshold, for which, as above, we refer the reader to the section ‘Mosaics and Y Chromosome Structural Anomalies’.

## 7. The MSY Euchromatic (MSYe) Region

### 7.1. Overview of the MSYe Region

**Figure 2:**
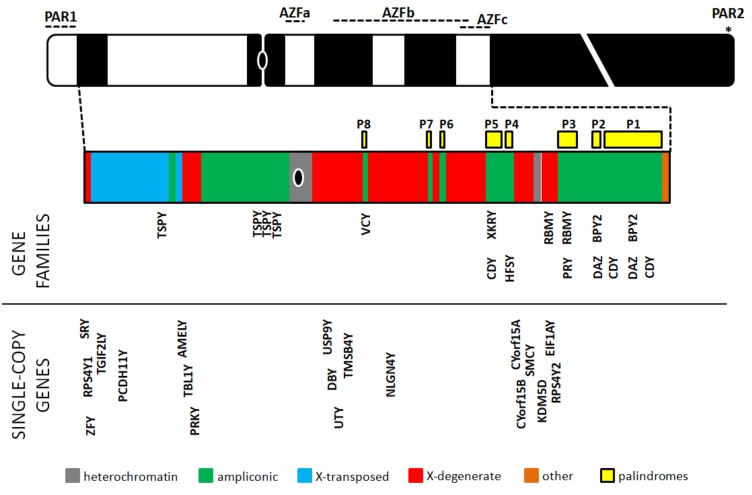
Organization of the euchromatic portion of the MSY region (MSYe). The picture also reports the localization of the palindromic regions P1–P8 and, for comparison with Figure 1, also PARs and AZF regions. The black oval indicates the centromere. The region q12 is shortened (white cut), due to its large extension compared to the rest of the chromosome. The colored MSYe region is slightly enlarged compared to the Y chromosome (grey lines) to enhance the readability. Gene families and single-copy genes are reported according to [128], with minor modifications.

The MSY region (euchromatin plus heterochromatin) (Figure 1) represents 95% of the Y chromosome (the remaining 5% are the PAR regions) and is responsible for important biological processes, such as sex determination and male fertility. Therefore, several scientists investigated MSY role in traits and diseases, such as sex determination and reversal, spermatogenesis and male infertility, urogenital malignancies, such as prostate cancer, sex-specific effects on the brain and behavior, male viability and graft-versus-host disease [20,113,119]; in some cases, these genes also show a dosage-dependent function with their X-linked counterparts [20]. Most recently, MSY has also been studied with high-throughput techniques [120,121]. Despite the lack of homology between the X chromosome and the MSY region, recent evidence demonstrates that a specialized form of recombination, namely gene conversion [122], may take place in human MSY [123,124,125,126], and that these events occur (i) within the Y chromosome (intrachromosomal recombination by gene conversion or other mechanisms), (ii) between the Y and X chromosomes, and also, although to a lesser extent, (iii) between the Y chromosome and some autosomal sequences [127]. Inside the MSY region, there is a mosaic of heterochromatic sequences, mostly composed of satellite DNA, alternating with three main euchromatic sequences: The X-transposed, X-degenerate and ampliconic regions [128] (Figure 2). The X-transposed sequences are 99% identical to DNA sequences in Xq21; their presence in the human MSY is the result of a massive X-to-Y transposition that occurred about 3–4 million years ago, after the divergence of the human and chimpanzee lineages [129]. Having a combined length of 3.4 Mb, the X-transposed sequences exhibit the lowest density of genes among the three aforementioned sequence classes of the MSY euchromatin, only encoding for two genes [130]; the rest of these regions is made of interspersed, repeated elements [128]. The X-degenerate sequences display single-copy genes or pseudogenes, homologues of 27 different X-linked genes, having between 60% and 96% nucleotide sequence identity with their X-linked counterparts. These regions probably consist of the remnants of the ancient autosomes from which the modern X and Y chromosomes evolved, and the single-copy genes they harbor show remarkable evolutionary conservation [131]. They have an 8.6 Mb total length and contain 16 coding genes, 12 of which are ubiquitously expressed; on the other hand, out of the 11 MSY genes, which are predominantly expressed in testis, only one, the *Sex-determining Region Y* (*SRY*), is located in the X-degenerate region [132]. Finally, the highly dynamic [43,44,133,134,135] ampliconic region has a length of 10.2 Mb, i.e., about 30% of the MSY euchromatin. It is largely composed of sequences that are very similar (more than 99.9%) to other MSY sequences; this consistency is maintained by frequent gene conversion and, despite their ample variability between species, their total amount is stably maintained throughout human lineages [121,136]. In this region there are eight massive palindromes, at least six of which contain testis-specific genes; therefore, the ampliconic region has the highest density of genes, both coding and non-coding, among the three sequence regions in the MSY euchromatin, resulting in 9 protein-coding gene families, which are responsible for 60 transcripts [137]. The content of the MSY region is highly dynamic; the MSY-associated CNV (amplifications and deletions) repeatedly happened during human evolution, at a very fast rate and in recent times. However, the overall amount of these sequences falls within a limited range even in geographically distant populations [25,138].

### 7.2. Spermatogenesis-Related Genes in the Yp

Some genes mapping in the short arm of the Y chromosome (Yp) are expressed in testes, but often this is not a specific signature; in addition, the role of some of these genes in the spermatogenesis is currently unclear [21,104]. Here we recall two of these genes, for the sake of completeness (Figure 2). *Zinc finger protein, Y-linked (ZFY*) is expressed in germline, and Leydig cells [139], and mice knockout for *Zfy* genes are infertile [140]; in addition, its function in somatic cells seems minor, if any [141]. Nonetheless, the lack of known mutations of this gene alone in infertile men does not allow confirming what it does in human spermatogenesis. *Testis-specific protein Y-linked (TSPY*) is considered a proto-oncogene that antagonizes its X-linked homologue (*TSPX*) [142]. To date, the function of *TSPY* in the spermatogenesis is controversial [21]: Some authors hypothesize that it might act as a pro-proliferative factor in a dosage-dependent manner [143,144]; however, recently this view has been questioned [145] suggesting a possible population stratification bias [21]. Consequently, no strong evidence exists to date of a relation between Yp CNV and male infertility.

### 7.3. Yq Chromosome Microdeletions and the Azoospermia Factor (AZF) Regions

Most genes mapping in the Y chromosome belong to its euchromatic portion, and several of those directly involved in the spermatogenesis are in the proximal part of its long arm (Yq11). Three distinct regions have been described in this arm, namely AZFa, AZFb and AZFc [146] (Figure 1 and Figure 2); a putative fourth region (AZFd) that was hypothesized by Kent-First and co-workers [147] does not exist [148]. Although AZFs harbor also functions that are not specifically required for fertility [119,149,150], their loss is usually associated with azoospermia or severe oligoasthenozoospermia and, consequently, to infertility. Indeed, AZF microdeletions are the most frequent structural chromosomal abnormalities and the major cause of CNV-related male infertility: They occur in approximately 10–15% of azoospermic and 5–10% of severe oligospermic patients [21,151,152]. However, the phenotype associated with different AZF microdeletions may significantly vary among patients and increasing evidence points towards population-based biases [21]. Microdeletions may span one or more AZF regions, or be partial deletions of two contiguous AZF regions; in addition, because some genes inside these regions are present in multiple copies, the extent of gene families’ deletions may influence the final phenotype [21,146,153,154], and assessing a direct relationship between their CNV and male fertility is sometimes problematic. The possibility that microdeletions of the AZF regions may cause other pathologies than infertility is still a matter of research; studies performed on both mosaic (45,XX/46,XY) and non-mosaic men suggest that this could be true at least for some specific microdeletions, but the scarcity of available data strongly limits what we know about this matter [21] (and references therein). All known AZF microdeletions are due to errors in the intrachromosomal recombination process [21,155,156,157].

The molecular analysis of AZF loci allowed the identification of several genes having a direct role in the spermatogenesis, thus, explaining infertility of men bearing microdeletions of these sequences (Figure 2).

In AZFa at least three loci, possibly important for male fertility, were identified, *Ubiquitin specific peptidase 9, Y-linked (USP9Y), Dead box on Y (DBY)* and *Ubiquitously transcribed tetratricopeptide repeat gene, Y-linked (UTY). USP9Y*, also known as *DFFRY*, is a protein-coding gene located on Yq11.221; it is a member of the peptidase C19 family and consists of 46 exons spanning 159 kb of genomic DNA [158]. This gene encodes a protein that is similar to ubiquitin-specific proteases, which cleaves the ubiquitin moiety from ubiquitin-fused precursors and ubiquitylated proteins. The USP9Y protein may play an important role at the level of protein turnover by preventing degradation of proteins by the proteasome through the removal of ubiquitin from protein–ubiquitin conjugates [159]. Considerable evidence has been collected during the past years, linking several kinds of mutations of the USP9Y gene to male infertility, especially to diseases like SPGFY2 and Y Chromosome Infertility [160], possibly by stabilizing a specific target protein that is important for male germ cell development [161]. However, the discovery of fertile men in a family with this locus deletion [162] has shown that *USP9Y* mutations alone are insufficient to cause infertility. Instead, *DBY* is very important for the spermatogenesis [163], since it is specifically expressed in the spermatogonia [164], and is frequently mutated/deleted in infertile men. The *UTY* deletion has been associated with male infertility [163], and some of its missense mutations have deleterious effects on the spermatogenesis [165]. However, because no cases of *UTY* deletion alone have been identified, the role played by this gene in normal spermatogenesis remains to be fully understood.

AZFb contains both single copy genes and gene families. Among single copy genes, the most interesting for male fertility are *lysine (K)-specific demethylase 5D* (*KDM5D)* and *Ribosomal protein S4, Y isoform 2* (*RPS4Y2)*. *KDM5D* encodes a histone H3 lysine 4 (H3K4) demethylase that is probably involved in male germ cell chromatin remodeling [166,167]. *RPS4Y2* is a protein-coding gene located on Yq11.223. This gene encodes a ribosomal protein specifically expressed in testis, being in contrast with the Y-linked *RPS4Y1* (located in the short arm of the Y) and the X-linked *RPS4X*, which are ubiquitously expressed paralogues [168]. One of the Y-linked copies of the Ribosomal Protein S4 is preferentially expressed during the spermatogenesis and might be important for germ cell development: In fact, the exclusive expression pattern of RPS4Y2 may indicate the functional role of this protein in posttranscriptional regulation of spermatogenesis [169,170]. As for gene families, potential candidates for fertility control are *XK, Kell blood group complex subunit-related, Y-linked* (*XKRY)*, *heat shock transcription factor, Y-linked* (*HSFY)*, *RNA binding motif protein, Y-linked, family 1, member A1* (*RBMY1A1),* and *PTPN13-like, Y-linked* (*PRY*). *XKRY* is located at position Yq11.222. Its encoded protein is called Testis-specific XK-related protein, Y-linked, which is similar to XK (X-linked Kell blood group precursor), a putative membrane transport protein. Dysfunctions or deletions of the XKRY gene are associated with spermatogenic failure and male infertility. *XKRY* shows a testis-specific expression [171,172], but its contribution to male spermatogenesis is considered marginal as its deletion is heritable [173]. *RBMY1A1*, located on Yq11.223, encodes for a male germ cell-specific RNA-binding protein associated with spermatogenesis. *RBMY1A1* has a testis-specific expression [174,175]; its role in the AZFb deletion-related phenotype has been described [176,177]. This protein is thought to function as a splicing regulator during spermatogenesis and microdeletions of the *RBMY* gene family are strongly associated with male infertility [177]. *RBMY* has also a homologue on the X-chromosome, named *RBMX*: RBMX and RBMY are members of an ancient pair of genes located on the sex chromosomes that encode RNA-binding proteins involved in the splicing. These genes have differentiated and evolved separately: *RBMY* has acquired a testis-specific function, while *RBMX* is ubiquitously expressed [178]. To investigate the number of functional genes, *RBM* expression has been studied through RT-PCR of *RBM* transcripts and characterizing *RBM* cDNA clones from six *RBM* subfamilies; of them, only the *RBMI* subfamily is actively transcribed. A total of six *RBMI* genes were identified, which produce four polypeptides [179]. *RBMY1A1* CNV is associated with sperm motility, i.e., its low copy number (<6) increases the risk of asthenozoospermia [180]. *HSFY* belongs to the heat shock factor family that has been shown to be implicated in spermatogenesis both in animals and humans. This gene maps at position Yq11.222. It has been discovered that two identical and functional full-length copies of *HSFY* map inside the palindrome P4, whereas, four similar sequences mapping in two clusters in palindrome P1 of AZFc and P3 seem to represent pseudogenes [181]. Deletions of this gene may be involved in azoospermia or oligospermia [182], suggesting its implication in unexplained cases of idiopathic male infertility [183]. More precisely, it has been shown that alterations of spermatogenic cell differentiation may be associated with altered expression of HSFY in the testis, leading to deteriorated spermatogenesis processes [184]. Overall *HSFY* is another strong candidate for infertility issues, since (i) it is expressed in testes, especially in round spermatids [182,185], (ii) its protein levels are low in spermatogenic cell samples from patients showing maturation arrest [184,186], and (iii) one azoospermic patient has been described, who had a small AZFb deletion, including only the two copies of *HSFY* [183]. Nevertheless, this interpretation is not final and has been challenged by some groups [173]. *PRY* is a protein-coding gene family, which has been proposed as a candidate spermatogenesis effector because of its essential role in the regulation of apoptosis [187]. It is located at position Yq11.223 and is specifically expressed in testis. Five exons were identified, by comparison of the cDNA sequence with the genomic sequence: Therefore, the presence of two full-length copies in AZFb (*PRY1* and *PRY2*) and two shorter versions of the *PRY* gene containing exons 3, 4 and 5 in AZFc (*PRY3* and *PRY4*) were discovered analyzing GenBank-derived clones on the Y chromosome [188]. *PRY* encodes a protein, which has a low degree of similarity with protein tyrosine phosphatase, non-receptor type 13 (PTPN13). It is expressed in testicular tissue and in the ejaculated sperm; furthermore, immunocytochemistry on testicular tissue showed the expression of the *PRY* gene in a small number of spermatozoa and spermatids. In the male ejaculate of 18 infertile couples, the PRY protein was found in 1.5–51.2% of spermatozoa and in most of the sperm precursor cells, suggesting its involvement in apoptosis of spermatids and spermatozoa [189]. Large deletions of AZFb, including *PRY1* and *PRY2*, seem to cause complete meiotic arrest, which leads to male infertility [190], whereas, patients with deletions of all the AZFb genes, except *PRY* and *RBMY*, display hypo-spermatogenesis [191]; these data validate *RBMY* and *PRY* central importance in male fertility processes [192]. For some authors, *PRY* might be an important infertility marker, more than a cause of infertility *per se*. In fact, its expression is irregular and restricted to few cells in the normal germ line, but the PRY protein is typically over-expressed in sperms having DNA fragmentation [188], which shows its apoptotic involvement [189].

Finally, inside the AZFc region, the candidate genes for infertility are *Chromodomain Y, Y-linked* (*CDY)*, *Basic charge, Y-linked, 1* (*BPY1*), *Basic protein Y2, Y-linked* (*BPY2)* and *Deleted in Azoospermia* (*DAZ)*. *CDY* is a human Y-chromosomal gene family expressed exclusively in the testis and implicated in male infertility; it is located at position Yq11.23. Its protein is localized in the nucleus of late spermatids, where histone hyperacetylation takes place; in fact, CDY protein has histone acetyltransferase activity, with a preference for histone H4 [193]. Since histone hyperacetylation is thought to facilitate the transition in which protamines replace histones as the major DNA-packaging protein, this feature offers a plausible mechanism to account for spermatogenic failure in patients bearing deletions of the *CDY* genes [194]. A strong association exists between loss of the *CDY1a* sequence family variant and male infertility (*p* = 0.002); overall, this genetic alteration, due to gene deletion or conversion, may represent a major risk factor for male infertility [195]; a similar scenario has been described for *CDY1b* [196]. *VCY*, also known as *Basic Protein Y 1* (*BPY1*), is a multi-copy gene family located on Yq11.221. This gene family has multiple members on both X and Y: Members of the VCX and VCY family share a high degree of sequence identity, except for a 30-nucleotide unit that is tandemly repeated in X-linked members, but is present only once in Y-linked members [171]. Therefore, all these members are expressed exclusively in male germ cells, especially in the nuclei of germ cells of the seminiferous epithelium [197]. The protein encoded by this gene is called Testis-specific basic protein Y 1 and is found only in the testicular tissue; it may be involved in the spermatogenesis or play a role in sex ratio distortion; however, its direct role in male fertility, if any, is currently unknown. Interestingly, it has been shown by a yeast two-hybrid assay that VCY interacts with acidic ribosomal protein P0, suggesting its possible involvement in the regulation of ribosome assembly during spermatogenesis [197]. *BPY2* is a protein-coding gene mapping in Yq11.223; it belongs to the *VCX/VCY* gene family as well, although it is not related to *BPY1* [198], and it is also known as *VCY2*. *BPY2* contains eight exons, with the initiating ATG codon in exon 4; its encoded protein (Testis-specific basic protein Y 2) interacts with ubiquitin protein ligase E3A and may be involved in male germ cell development and male infertility—in fact, its deletions are frequently found in infertile men with severe oligozoospermia or azoospermia [199]. *BPY2* possible role in male infertility upon duplication has been suggested as well [200]. *BPY2* encodes a highly charged testis-specific protein that localizes to the nucleus of all-male germ cells [197,201]. Immunohistochemical analyses showed that *BPY2* localized to the nuclei of spermatogonia, spermatocytes, and round spermatids, but not in elongated spermatids. At the ultrastructural level, *VCY2* was expressed in the nucleus of human ejaculated spermatozoa [201]. Although *VCY* role in the spermatogenesis is unclear, some authors suggest its potential involvement in the cytoskeletal regulation of the spermatogenesis, based on its interaction with a protein (namely, variable charge, Y chromosome 2 interacting protein-1 (VCY2IP-1), also known as MAP1S) having a high degree of homology with the Microtubule-Associated Proteins (MAPs) MAP1A and MAP1B [202]. *DAZ*, of autosomic origin [203], is a multicopy gene family mapping at Yq11.223 and is organized in a repeated cluster containing four gene copies (*DAZ1-4*). Each gene copy contains at least seven tandem copies of a 2.4-kb repeat unit that encodes 24 amino acids, with DAZ1 paired with DAZ2, and DAZ3 paired with DAZ4 [204]. The DAZ proteins are all encoded by a strongly repeated region of the Y chromosome and appear to be very similar to each other; thus, it is very likely that the described interactions with any DAZ protein may involve all four proteins. DAZ genes belong to a greater family of genes, which includes also BOULE (or BOLL, boule-like) and DAZL (deleted in azoospermia-like) genes that map on autosomes and may serve as *backup genes*, which would help to preserve a residual spermatogenesis in males with AZFc deletions that remove the Y-linked DAZ genes [205]. Complete deletions of DAZ genes have been associated with severe disruption of spermatogenesis [206], and might be one of the causes of a condition called Spermatogenic failure Y-linked 2 (SPGFY2) (OMIM ID: **#** 415000). The four *DAZ* genes are expressed in the spermatogonia and encode for 3’-UTR RNA-binding proteins, which regulate the RNA translation during the germline cell progression to meiosis and the formation of haploid germ cells [207,208]. The proofs of the *DAZ* pivotal function in spermatogenesis are several, since its protein localizes in the late spermatids and spermatozoa tails [209,210], and the frequency of its deletion in infertile men is high [211]. However, the role of the *DAZ* gene deletion alone in the spermatogenesis impairment, without additional genetic alterations, has been questioned [118,212]. Besides the well-known spermatogenesis impairment caused by *DAZ* genes loss, some research indicates that also a primary duplication of *DAZ* genes may cause male fertility disorders [200,213,214,215,216]. This suggests that this gene family CNV (and, possibly, the total amount of copies of genes found in the AZFc region, such as *BPY2* and *CDY1*) might be under strict genetic control. These observations about *DAZ* and *BPY2* genes might—at least partially—explain the impaired fertility of men with duplication/hyperploidy of the Y chromosome without recognized, specific gene mutations, i.e., men with supernumerary, wild type, Y-linked DNA sequences (see the sections ‘Mosaics and Y Chromosome Structural Anomalies’ and ‘Y Chromosome Aneuploidies’).

## 8. The MSY Heterochromatic Region (MSYh)

The distal portion of Yq, mapping at Yq12 and totaling roughly 40Mb [128], excluding the PAR2 region (Figure 1), is essentially composed of constitutive heterochromatin and mainly contains two highly repetitive families of satellite sequences, called DYZ1 and DYZ2, made up of about 5000 and 2000 copies of each satellite, respectively [23,217]. These DYZ regions account for approximately 50% of the total Y chromosome DNA [218]. Patients with either longer or shorter MSY have been reported [219,220,221] (and references therein) and, to a certain extent, these differences fall within the normal Y-length polymorphisms [222,223]. However, in many cases either this is not the only karyotype anomaly or patients are mosaics for such alterations; thus, discriminating the role of Y-linked aberrations in the patients’ phenotype—if any—is difficult. In addition, according to some authors, even in cytogenetically Yq negative individuals, it is possible to detect residual DYZ1 sequences (as short as 3.4Kb) using suitable probes, and this condition is still compatible with fertility [224]. Similarly, DYZ2 residual sequences were detected in samples from patients with small Y chromosomes [225], but not from others having the same anomaly [217]. There are chances that residual copies of both DYZ1 and DYZ2 were also present in samples classified as negatives, but they remained undetected because of the relatively low resolution of the used techniques (Southern blot, cytogenetics, restriction enzyme digestion). Altogether, these data suggest a minor role, or no role at all, for DYZs in male development, also considering that the MSY heterochromatic region may be present in healthy females as a consequence of Y-autosome translocations [226]. However, others suggest a role at least for DYZ1. According to these authors, DYZ CNV may be connected with recurrent spontaneous abortion and early embryo growth arrest both when DYZ repeats significantly increase or decrease [227]; moreover, a reduced number of copies of DYZ1 was found in infertile men, although a clear cause-effect relationship is missing [118]. Reports describing variations of the MSYh region are rare [221,228]; MSYh evolution is relatively recent and probably started after speciation of humans from their common ancestor [229]. Indeed, variations in the amount of DYZ sequences have been reported, and their estimated frequency seems higher than for similar events in the rest of the genome [230]. However, in all described cases, the variation does not go beyond a few repeated units, and the size variation of the Y chromosome is, on average, quite limited. Therefore, the MSYh region might be under evolutionary pressure, either because it fulfils a yet unknown function and/or because there are mechanisms that preserve its length within a certain range. It is useful to recall here the study of Jehan and collaborators [231], who found a developmental stage- and tissue-specific transcription of DYZ1 at week 36 of pregnancy and in adults, inside the testis. This DYZ1 transcript has a stretch of 67 nucleotides, which almost perfectly match (66/67) the 5’UTR of *CDC2L2 β sv13* isoform mapping in 1p36.3. In addition, the DYZ1 transcript found in the testis is chimeric and originated by *trans*-splicing [232], joining the DYZ1 homologous stretch with the *CDC2L2 β sv13* coding sequence; this transcript is testis-specific, while the transcript from the chromosome 1 is found in the brain, but not in the testes. The functional role of the DYZ1 RNA is unclear.

Other possible clues about the MSYh role in the human genome come from a 30 year old report by Nazarenko and co-workers [233]. They studied 55 men with an almost complete deletion of the MSYh region and reported that the Y-linked heterochromatin has a phenotypic effect on a significant number (20 out of the 80 analyzed) of quantitative anthropometric features. Other reports propose a link between excess of total genomic heterochromatin and male infertility, including both autosomal (chromosomes 1, 9, 16, 18) and Y-linked heterochromatin [234] (and references therein). Altogether, these articles suggest that there are both upper and lower limits to the Y-linked heterochromatin length, with (i) excessive heterochromatin possibly causing (or participating to some extent to) male infertility and (ii) insufficient heterochromatin causing various morphological and physiological changes. Some authors propose that epigenetic regulation may involve the Y chromosome heterochromatin, with DNA-binding chromatin regulators sequestered to the lengthy Y chromosome heterochromatic domains [235]. This would resemble what happens in other organisms, such as *Drosophila melanogaster*, where polymorphic variations of the Y chromosome heterochromatin result in genome-wide gene expression variations [236]. It is possible to hypothesize that the CNV of the Y-linked heterochromatic sequences, in combination with other heterochromatic, autosomal sequences, have a quantitative role on gene expression. It would follow that the total amount of genomic constitutive heterochromatin might influence the transcription of target genes by indirectly acting as a repository for chromatin-modifying proteins, which in turn may affect male fertility in specific genetic backgrounds.

## 9. Discussion

Male fertility is determined by several, complex, interconnected events that include the action of single genes (mapping in different genome locations, such as *SOX9* or *AR*), the activity of gene families (e.g., those in the *AZF* loci), and epigenetic factors, such as (1) DNA modifications, (2) non-coding RNAs altering gene expression or acting via *trans*-splicing, and (3) constitutive heterochromatin that may interfere with the availability of transcription factors and/or chromatin-modifying proteins. Available data support the idea that both qualitative and quantitative factors have a role in these events and considering any one of them, without accounting for the others, is insufficient, sometimes, to explain male fertility impairment. Single point mutations or gene deletions might be identified as possible causes of spermatogenesis failure in many idiopathic patients; however, in many others, while the wild type gene sequences are preserved, their total amount is altered. CNV are one of the major causes of human genome variability and, essentially, they are the deletion or the duplication of the original sequence (or parts of it), without any additional modification (mutation), consequent to unequal crossing-over between or within chromosomes. CNV are frequently associated to both Mendelian and more complex conditions and are based on several, different molecular events, such as gene disruption, gene fusion and gene expression modification consequent to chromosome sequence relocation [237]. CNV may have positive consequences for species, from an evolutionary perspective, being the basis for the acquisition of new genetic functions after gene duplication or exon shuffling [237]. However, at an individual level, growing evidence supports the existence of loci that may impair male fertility as a consequence of CNV and which map on the autosomes, as well as on the X chromosome [238,239,240,241,242,243]. Here we recapitulate results that Y-linked genes may affect male fertility through the same mechanism.

We have discussed structural and/or numerical anomalies of the Y chromosome that may deeply influence the male fertility, independently of the sequence of protein-coding genes harbored in this chromosome. Indeed, some genes mapping on the Y chromosome have a direct role in male fertility [104], so that any chromosomal anomaly (deletions, some unbalanced translocations) causing underexpression or loss of such functions is able to impair the male ability to procreate. In this perspective, the *SRY* gene and *AZF* loci can be seen as models. Much less clear is the situation when there are additional copies of one or more genes, as in aneuploidies, duplications or some unbalanced translocations, having a wild type sequence. For some Y-linked genetic functions (such as *SMCY*), it has been shown a dosage-dependent fertility impairment, although not directly related to the male physiology. However, there are also studies supporting a direct relationship between CNV of other genes—such as *RBMY1* and DAZ—and male sterility. Accordingly, it is possible to hypothesize that other male fertility-related Y-linked genes are under dosage control as well. Indeed, for some of them, either an up- or down-regulation has been demonstrated in infertile men [244], although it is unclear if this is a cause or rather a consequence of infertility. While, for most of these genes, a dosage-dependent fertility impairment has not yet been established, it would be in good agreement with the escape from meiotic sex chromosome inactivation and related consequences on male fertility described by Royo and collaborators [76].

Sometimes, the structure of the Y chromosome is at least as important as its gene content, and it might even be more crucial than it. PAR1 does not harbor genes ‘specifically’ affecting male fertility, but its absence causes sex chromosomes mis-segregation and, consequently, infertility. PAR1 resembles ‘chromosome structural information’, like centromeres and telomeres, whose function relies on their structure, as well as on their sequence, as for the karyotype stability. Essentially, PAR1 does not promote male fertility through its gene content, but because of its ability to recombine with the X chromosome. Consequently, an increase in the number of PARs, as it occurs in sex chromosomes aneuploidies, could impair the ability of sex chromosomes to correctly pair and segregate in meiosis. The PAR2 deletion does not impair fertility, but patients lacking PAR2 have an intact PAR1 region. It would be interesting to check if cells with a deletion of PAR1, but a complete PAR2 are still able to correctly segregate the sex chromosomes in meiosis; of course, this could be tested in vivo only in mosaic men, because of the importance of PAR1 genes for patients’ health. Similarly, the apparent contradiction between the stability of the MSYh region length and its lack of protein-coding genes might underline some not yet understood role for the Y-linked constitutive heterochromatin as a whole, which is not linked to specific sequences contained in it, but is sufficient to put this region under evolutionary constraints preventing major variations of its size. The MSYh region is highly dynamic, being able to gain and lose sequences very quickly, yet the overall amount of MSYh-linked chromatin in different populations is, in most cases, relatively constant. Indeed, the few available data indicate that, in most cases, the Y chromosome multisomy causes severe sperm count impairment. This outcome fits quite well the findings of Yakin and collaborators, who described a correlation between total genomic heterochromatin and male fertility [234], suggesting that the Y chromosome might also influence the expression of (likely, several) genes involved in male fertility through epigenetic mechanisms. Further studies and more patients are necessary to validate this hypothesis.

## 10. Conclusions

The Y chromosome role in human physiology, despite the low number of its genes compared to other chromosomes of similar size, is far from simple. The genes harbored in Y are involved not only in male fertility, but also in several other functions, such as neurological activities, morphogenesis and development. In addition, it has been demonstrated that in most cases (excluding genes mapping in PARs) the genes of this chromosome do not behave in a Mendelian fashion and are influenced by the genetic background of the host organism. Epigenetic mechanisms are likely involved too, thus, further complicating the genetic background in which the Y chromosome operates. All these aspects possibly make the Y the most complicated chromosome with respect to human reproduction. In cases of male infertility where an evident, common cause of this inability is missing (e.g., point mutations of known genes or physiological/morphological conditions), a genetic analysis of the Y chromosome content is strongly advised, since the mere sequencing of fertility-related genes might not disclose karyotype anomalies, especially in the case of supernumerary, wild type copies of known genes or when there are alterations in its chromatin content/structure. Genes mapping on the Y chromosome act in a complex fashion and a strong effort is required to comprehensively understand their role and interaction with multiple genetic pathways.

## Figures and Tables

**Table 1:** Comparison between the traditional and newly proposed, clinically based classification of male infertility etiology.

Traditional Classification	Examples
Pre-testicular causes	Hypogonadotropic hypogonadism Pituitary diseases Coital disorders
Testicular causes	Varicocele Cryptorchidism Noonan Syndrome Vanishing Testis Syndrome Myotonic dystrophy 46,XX testicular disorders of sex development 47,XYY Syndrome Klinefelter’s Syndrome Y chromosome microdeletions Sertoli Cell-Only Syndrome (germ cell aplasia) Gonadotoxins Systemic diseases Testis injury Idiopathic infertility
Post-testicular causes	Male reproductive tract obstruction Disorders of sperm function or sperm motility Disorders of coitus
**Clinically Based Classification**	**Examples (Top: Genetic Causes; Bottom: Non-Genetic or Mixed Causes)**
Hypothalamic–pituitary axis	Kallmann’s Syndrome
	Ablative treatments (e.g., surgery or radiation); pituitary adenomas; tumors of the CNS; infection; infiltrative disease; empty sella syndrome; autoimmune hypophysitis; abuse of anabolic steroids; testosterone-replacement therapy; use or abuse of opiates and their analogues
Quantitative Spermatogenesis (i.e., affecting the production and numbers of sperm)	Gross chromosomal/karyotype anomalies; submicroscopic deletions (such as AZF deletions); Klinefelter’s Syndrome (47,XXY); 46,XX male syndrome; isodicentric Y chromosome; structural aberrations of the autosomes; X-linked genetic anomalies (such as *AR* or *TEX11* gene mutations)
	Varicocele; previous cytotoxic chemotherapy or radiotherapy; mumps; viral orchitis; testicular torsion; gonadal malignancy; severe scrotal trauma; some common medications; severe systemic illness; cryptorchidism
Qualitative Spermatogenesis (i.e., affecting the characteristics of sperm, such as motility, fertilization, and genetic competency)	Globozoospermia (e.g., *SPATA16*, *PICK1*, *DPY19L2* gene mutations); macrozoospermia (e.g., *AURKC* gene mutations); ageing
	Oxidative stress; inflammation; infection; autoimmune reaction against the spermatozoa; phospholipase C ζ deficiencies
Ductal obstruction or dysfunction	Congenital bilateral absence of the *vas deferens* (CBAVD) (e.g., *CFTR* gene mutations)
	Vasectomy; epididymal occlusion; previous inguinal hernia repair with inadvertent interruption or scarring of the *vasa*; spinal cord injury affecting ejaculation; retrograde ejaculation; erectile dysfunction; Young’s Syndrome

**Table 2:** Semen characteristics in normozoospermic men. Data is according to the World Health Organization reference values [9]. Data indicate lower reference limits, the fifth centiles (with 95% confidence intervals). Time to pregnancy (TTP) is defined as the number of months elapsed between stopping contraception to achieving a pregnancy [12].

Parameter	Value (Interval)
Time to pregnancy (TTP)	12 months (upper limit)
Semen volume	1.5 mL (1.4–1.7)
Total spermatozoa number	39 million per ejaculate (33–46)
Spermatozoa concentration	15 million per mL (12–16)
Vitality	58% live (55–63)
Progressive motility	32% (31–34)
Total motility (progressive + non-progressive)	40% (38–42)
Morphologically normal forms	4.0% (3.0–4.0)

## References

[1] World Health Organization (2016). Infertility Definitions and Terminology.

[2] Agarwal A., Mulgund A., Hamada A., Chyatte M.R. (2015). A unique view on male infertility around the globe. Reprod. Biol. Endocrinol..

[3] Dimitriadis F., Adonakis G., Kaponis A., Mamoulakis C., Takenaka A., Sofikitis N. (2017). Pre-Testicular, Testicular, and Post-Testicular Causes of Male Infertility.

[4] Tournaye H., Krausz C., Oates R.D. (2017). Novel concepts in the aetiology of male reproductive impairment. Lancet Diabetes Endocrinol..

[5] European Association of Urology (EAU) Guidelines—Male Infertility. http://uroweb.org/guideline/male-infertility/.

[6] Hamada A., Esteves S., Agarwal A. (2013). A comprehensive review of genetics and genetic testing in azoospermia. Clinics.

[7] Wosnitzer M.S., Goldstein M. (2014). Obstructive azoospermia. Urol. Clin. North Am..

[8] Barazani Y., Katz B.F., Nagler H.M., Stember D.S. (2014). Lifestyle, Environment, and Male Reproductive Health. Urol. Clin. North Am..

[9] Cooper T.G., Noonan E., von Eckardstein S., Auger J., Baker H.W.G., Behre H.M., Haugen T.B., Kruger T., Wang C., Mbizvo M.T. (2010). World Health Organization reference values for human semen characteristics. Hum. Reprod. Update.

[10] Levine H., Jørgensen N., Martino-Andrade A., Mendiola J., Weksler-Derri D., Mindlis I., Pinotti R., Swan S.H. (2017). Temporal trends in sperm count: A systematic review and meta-regression analysis. Hum. Reprod. Update.

[11] Johnson S.L., Dunleavy J., Gemmell N.J., Nakagawa S. (2015). Consistent age-dependent declines in human semen quality: A systematic review and meta-analysis. Ageing Res. Rev..

[12] Joffe M. (2000). Time trends in biological fertility in Britain. Lancet.

[13] Bachtrog D. (2013). Y-chromosome evolution: Emerging insights into processes of Y-chromosome degeneration. Nat. Rev. Genet..

[14] Ross M.T., Grafham D.V., Coffey A.J., Scherer S., McLay K., Muzny D., Platzer M., Howell G.R., Burrows C., Bird C.P. (2005). The DNA sequence of the human X chromosome. Nature.

[15] Hughes J.F., Page D.C. (2015). The Biology and Evolution of Mammalian Y Chromosomes. Annu. Rev. Genet..

[16] Bellott D.W., Page D.C. (2009). Reconstructing the Evolution of Vertebrate Sex Chromosomes. Cold Spring Harb. Symp. Quant. Biol..

[17] Gulía C., Baldassarra S., Zangari A., Briganti V., Gigli S., Gaffi M., Signore F., Vallone C., Nucciotti R., Costantini F.M. (2018). Androgen insensitivity syndrome. Eur. Rev. Med. Pharmacol. Sci..

[18] Hallast P., Jobling M.A. (2017). The Y chromosomes of the great apes. Hum. Genet..

[19] Hughes J.F., Skaletsky H., Koutseva N., Pyntikova T., Page D.C. (2015). Sex chromosome-to-autosome transposition events counter Y-chromosome gene loss in mammals. Genome Biol..

[20] Bellott D.W., Hughes J.F., Skaletsky H., Brown L.G., Pyntikova T., Cho T.-J., Koutseva N., Zaghlul S., Graves T., Rock S. (2014). Mammalian Y chromosomes retain widely expressed dosage-sensitive regulators. Nature.

[21] Krausz C., Casamonti E. (2017). Spermatogenic failure and the Y chromosome. Hum. Genet..

[22] Graves J.A. (1995). The origin and function of the mammalian Y chromosome and Y-borne genes—An evolving understanding. Bioessays.

[23] Quintana-Murci L., Fellous M. (2001). The Human Y Chromosome: The Biological Role of a Functional Wasteland. J. Biomed. Biotechnol..

[24] Poznik G.D., Xue Y., Mendez F.L., Willems T.F., Massaia A., Wilson Sayres M.A., Ayub Q., McCarthy S.A., Narechania A., Kashin S. (2016). Punctuated bursts in human male demography inferred from 1,244 worldwide Y-chromosome sequences. Nat. Genet..

[25] Lucotte E.A., Skov L., Jensen J.M., Macià M.C., Munch K., Schierup M.H. (2018). Dynamic Copy Number Evolution of X- and Y-Linked Ampliconic Genes in Human Populations. Genetics.

[26] Naqvi S., Bellott D.W., Lin K.S., Page D.C. (2018). Conserved microRNA targeting reveals preexisting gene dosage sensitivities that shaped amniote sex chromosome evolution. Genome Res..

[27] Bellott D.W., Skaletsky H., Cho T.-J., Brown L., Locke D., Chen N., Galkina S., Pyntikova T., Koutseva N., Graves T. (2017). Avian W and mammalian Y chromosomes convergently retained dosage-sensitive regulators. Nat. Genet..

[28] San Roman A.K., Page D.C. (2019). A strategic research alliance: Turner syndrome and sex differences. Am. J. Med. Genet. C. Semin. Med. Genet..

[29] Haseltine P., Michael E., McClure M., Goldberg E. (1987). Genetic Markers of Sex Differentiation.

[30] Layman L.C., Tho S.P.T., Clark A.D., Kulharya A., McDonough P.G. (2009). Phenotypic spectrum of 45,X/46,XY males with a ring Y chromosome and bilaterally descended testes. Fertil. Steril..

[31] Reindollar R.H., Byrd J.R., Hahn D.H., Haseltine F.P., McDonough P.G. (1987). A cytogenetic and endocrinologic study of a set of monozygotic isokaryotic 45,X/46,XY twins discordant for phenotypic sex: Mosaicism versus chimerism. Fertil. Steril..

[32] Quilter C., Nathwani N., Conway G., Stanhope R., Ralph D., Bahadur G., Serhal P., Taylor K., Delhanty J. (2002). A comparative study between infertile males and patients with Turner syndrome to determine the influence of sex chromosome mosaicism and the breakpoints of structurally abnormal Y chromosomes on phenotypic sex. J. Med. Genet..

[33] Reddy K.S., Sulcova V. (1998). Pathogenetics of 45,X/46,XY gonadal mosaicism. Cytogenet. Genome Res..

[34] Beaulieu Bergeron M., Brochu P., Lemyre E., Lemieux N. (2011). Correlation of intercentromeric distance, mosaicism, and sexual phenotype: Molecular localization of breakpoints in isodicentric Y chromosomes. Am. J. Med. Genet. A.

[35] Gantt P.A., Byrd J.R., Greenblatt R.B., McDonough P.G. (1980). A clinical and cytogenetic study of fifteen patients with 45,X/46XY gonadal dysgenesis. Fertil. Steril..

[36] Tho S.P., Layman L.C., Lanclos K.D., Plouffe L., Byrd J.R., McDonough P.G. (1992). Absence of the testicular determining factor gene SRY in XX true hermaphrodites and presence of this locus in most subjects with gonadal dysgenesis caused by Y aneuploidy. Am. J. Obstet. Gynecol..

[37] Telvi L., Lebbar A., Del Pino O., Barbet J.P., Chaussain J.L. (1999). 45,X/46,XY mosaicism: Report of 27 cases. Pediatrics.

[38] Ly P., Teitz L.S., Kim D.H., Shoshani O., Skaletsky H., Fachinetti D., Page D.C., Cleveland D.W. (2017). Selective Y centromere inactivation triggers chromosome shattering in micronuclei and repair by non-homologous end joining. Nat. Cell Biol..

[39] Ghezraoui H., Piganeau M., Renouf B., Renaud J.-B., Sallmyr A., Ruis B., Oh S., Tomkinson A.E., Hendrickson E.A., Giovannangeli C. (2014). Chromosomal Translocations in Human Cells Are Generated by Canonical Nonhomologous End-Joining. Mol. Cell.

[40] Henegariu O., Pescovitz O.H., Vance G.H., Verbrugge J., Heerema N.A. (1997). A case with mosaic di-, tetra-, and octacentric ring Y chromosomes. Am. J. Med. Genet..

[41] Arnedo N., Nogués C., Bosch M., Templado C. (2005). Mitotic and meiotic behaviour of a naturally transmitted ring Y chromosome: Reproductive risk evaluation. Hum. Reprod..

[42] Schellberg R., Schwanitz G., Schweikert H.-U., Raff R. (2002). Chromosome Mosaicism in Patients with Normal and Abnormal Y-Chromosome. Int. J. Hum. Genet..

[43] Lange J., Skaletsky H., van Daalen S.K.M., Embry S.L., Korver C.M., Brown L.G., Oates R.D., Silber S., Repping S., Page D.C. (2009). Isodicentric Y Chromosomes and Sex Disorders as Byproducts of Homologous Recombination that Maintains Palindromes. Cell.

[44] Lange J., Noordam M.J., van Daalen S.K.M., Skaletsky H., Clark B.A., Macville M.V., Page D.C., Repping S. (2013). Intrachromosomal homologous recombination between inverted amplicons on opposing Y-chromosome arms. Genomics.

[45] Hsu L.Y. (1994). Phenotype/karyotype correlations of Y chromosome aneuploidy with emphasis on structural aberrations in postnatally diagnosed cases. Am. J. Med. Genet..

[46] Hughes I.A., Houk C., Ahmed S.F., Lee P.A., LWPES/ESPE Consensus Group (2005). Consensus statement on management of intersex disorders. Arch. Dis. Child..

[47] De la Chapelle A. (1972). Analytic review: nature and origin of males with XX sex chromosomes. Am. J. Hum. Genet..

[48] De la Chapelle A. (1981). The etiology of maleness in XX men. Hum. Genet..

[49] Ergun-Longmire B., Vinci G., Alonso L., Matthew S., Tansil S., Lin-Su K., McElreavey K., New M.I. (2005). Clinical, hormonal and cytogenetic evaluation of 46,XX males and review of the literature. J. Pediatr. Endocrinol. Metab..

[50] Vorona E., Zitzmann M., Gromoll J., Schüring A.N., Nieschlag E. (2007). Clinical, endocrinological, and epigenetic features of the 46,XX male syndrome, compared with 47,XXY Klinefelter patients. J. Clin. Endocrinol. Metab..

[51] Andersson M., Page D.C., de la Chapelle A. (1986). Chromosome Y-specific DNA is transferred to the short arm of X chromosome in human XX males. Science.

[52] Morales C., Soler A., Bruguera J., Madrigal I., Alsius M., Obon M., Margarit E., Sánchez A. (2007). Pseudodicentric 22;Y translocation transmitted through four generations of a large family without phenotypic repercussion. Cytogenet. Genome Res..

[53] Uçan B., Özbek M., Topaloğlu O., Yeşilyurt A., Güngüneş A., Demirci T., Delibaşı T. (2013). 46,XX Male Syndrome. Turk. J. Endocrinol. Metab..

[54] Kusz K., Kotecki M., Wojda A., Szarras-Czapnik M., Latos-Bielenska A., Warenik-Szymankiewicz A., Ruszczynska-Wolska A., Jaruzelska J. (1999). Incomplete masculinisation of XX subjects carrying the SRY gene on an inactive X chromosome. J. Med. Genet..

[55] Abusheikha N., Lass A., Brinsden P. (2001). XX males without SRY gene and with infertility. Hum. Reprod..

[56] Rajender S., Rajani V., Gupta N., Chakravarty B., Singh L., Thangaraj K. (2006). SRY-negative 46,XX male with normal genitals, complete masculinization and infertility. Mol. Hum. Reprod..

[57] Mustafa O., Mehmet E. (2010). A 46, XX SRY—negative man with infertility, and co-existing with chronic autoimmune thyroiditis. Gynecol. Endocrinol..

[58] Vetro A., Dehghani M.R., Kraoua L., Giorda R., Beri S., Cardarelli L., Merico M., Manolakos E., Parada-Bustamante A., Castro A. (2015). Testis development in the absence of SRY: Chromosomal rearrangements at SOX9 and SOX3. Eur. J. Hum. Genet..

[59] Grinspon R.P., Rey R.A. (2016). Disorders of Sex Development with Testicular Differentiation in SRY-Negative 46,XX Individuals: Clinical and Genetic Aspects. Sex. Dev..

[60] Genetics Home Reference. https://ghr.nlm.nih.gov/.

[61] Kim I.W., Khadilkar A.C., Ko E.Y., Sabanegh E.S. (2013). 47,XYY Syndrome and Male Infertility. Rev. Urol..

[62] Jacobs P.A., Melville M., Ratcliffe S., Keay A.J., Syme J. (1974). A cytogenetic survey of 11,680 newborn infants. Ann. Hum. Genet..

[63] Hann M.C., Lau P.E., Tempest H.G. (2011). Meiotic recombination and male infertility: From basic science to clinical reality?. Asian J. Androl..

[64] Harton G.L., Tempest H.G. (2012). Chromosomal disorders and male infertility. Asian J. Androl..

[65] Tempest H.G., Homa S.T., Dalakiouridou M., Christopikou D., Wright D., Zhai X.P., Griffin D.K. (2004). The association between male infertility and sperm disomy: Evidence for variation in disomy levels among individuals and a correlation between particular semen parameters and disomy of specific chromosome pairs. Reprod. Biol. Endocrinol..

[66] Chatziparasidou A., Christoforidis N., Samolada G., Nijs M. (2015). Sperm aneuploidy in infertile male patients: A systematic review of the literature. Andrologia.

[67] Boisen E., Rasmussen L. (1978). Tremor in XYY and XXY men. Acta Neurol. Scand..

[68] Evans J.A., Jane A., Hamerton J.L., John L., Robinson A. (1991). March of Dimes Birth Defects Foundation. Children and young adults with sex chromosome aneuploidy: Follow-up, clinical, and molecular studies. Proceedings of the 5th International Workshop on Sex, Chromosome Anomalies held at Minaki, ON, Canada, 7–10 June 1989.

[69] Brown W.M. (1968). Males with an XYY sex chromosome complement. J. Med. Genet..

[70] Theilgaard A. (1984). A psychological study of the personalities of XYY- and XXY-men. Acta Psychiatr. Scand. Suppl..

[71] Abdel-Razic M.M., Abdel-Hamid I.A., ElSobky E.S. (2012). Nonmosaic 47,XYY syndrome presenting with male infertility: Case series. Andrologia.

[72] Blanco J., Egozcue J., Vidal F. (2001). Meiotic behaviour of the sex chromosomes in three patients with sex chromosome anomalies (47,XXY, mosaic 46,XY/47,XXY and 47,XYY) assessed by fluorescence in-situ hybridization. Hum. Reprod..

[73] Moretti E., Anichini C., Sartini B., Collodel G. (2007). Sperm ultrastructure and meiotic segregation in an infertile 47, XYY man. Andrologia.

[74] Rives N., Milazzo J.P., Miraux L., North M.-O., Sibert L., Mace B. (2005). From spermatocytes to spermatozoa in an infertile XYY male. Int. J. Androl..

[75] Wong E.C., Ferguson K.A., Chow V., Ma S. (2008). Sperm aneuploidy and meiotic sex chromosome configurations in an infertile XYY male. Hum. Reprod..

[76] Royo H., Polikiewicz G., Mahadevaiah S.K., Prosser H., Mitchell M., Bradley A., de Rooij D.G., Burgoyne P.S., Turner J.M.A. (2010). Evidence that meiotic sex chromosome inactivation is essential for male fertility. Curr. Biol..

[77] Linden M.G., Bender B.G., Robinson A. (1995). Sex chromosome tetrasomy and pentasomy. Pediatrics.

[78] Frühmesser A., Kotzot D. (2011). Chromosomal Variants in Klinefelter Syndrome. Sex. Dev..

[79] Tartaglia N., Ayari N., Howell S., D’Epagnier C., Zeitler P. (2011). 48,XXYY, 48,XXXY and 49,XXXXY syndromes: Not just variants of Klinefelter syndrome. Acta Paediatr..

[80] Sørensen K., Nielsen J., Jacobsen P., Rølle T. (1978). The 48,XXYY syndrome. J. Ment. Defic. Res..

[81] Tartaglia N., Davis S., Hench A., Nimishakavi S., Beauregard R., Reynolds A., Fenton L., Albrecht L., Ross J., Visootsak J. (2008). A new look at XXYY syndrome: Medical and psychological features. Am. J. Med. Genet. A.

[82] Shanmugam V.K., Tsagaris K.C., Attinger C.E. (2012). Leg ulcers associated with Klinefelter’s syndrome: A case report and review of the literature. Int. Wound J..

[83] Bloomgarden Z.T., Delozier C.D., Cohen M.P., Kasselberg A.G., Engel E., Rabin D. (1980). Genetic and endocrine findings in a 48,XXYY male. J. Clin. Endocrinol. Metab..

[84] Roche C., Sonigo C., Benmiloud-Tandjaoui N., Boujenah J., Benzacken B., Poncelet C., Hugues J.-N. (2014). Azoospermie et tétrasomie 48,XXYY: Quelle prise en charge de l’infertilité?. Gynécologie Obs. Fertil..

[85] Hunter H., Quaife R. (1973). A 48,XYYY male: A somatic and psychiatric description. J. Med. Genet..

[86] Shanske A., Sachmechi I., Patel D.K., Bishnoi A., Rosner F. (1998). An adult with 49,XYYYY karyotype: Case report and endocrine studies. Am. J. Med. Genet..

[87] Paoloni-Giacobino A., Lespinasse J. (2007). Chromosome Y polysomy: A non-mosaic 49,XYYYY case. Clin. Dysmorphol..

[88] Demily C., Poisson A., Peyroux E., Gatellier V., Nicolas A., Rigard C., Schluth-Bolard C., Sanlaville D., Rossi M. (2017). Autism spectrum disorder associated with 49,XYYYY: Case report and review of the literature. BMC Med. Genet..

[89] Van Den Berghe H., Verresen H., Cassiman J.J. (1968). Letters to the Editor: A Male with 4 Y-Chromosomes. J. Clin. Endocrinol. Metab..

[90] Bichile D., Kharkar A., Menon P., Potnis-Lele M., Bankar M., Shroff G. (2014). Y chromosome: Structure and biological functions. Indian J. Basic Appl. Med. Res..

[91] Dhanoa J.K., Mukhopadhyay C.S., Arora J.S. (2016). Y-chromosomal genes affecting male fertility: A review. Vet. World.

[92] Kauppi L., Barchi M., Baudat F., Romanienko P.J., Keeney S., Jasin M. (2011). Distinct properties of the XY pseudoautosomal region crucial for male meiosis. Science.

[93] Ellison J.W., Wardak Z., Young M.F., Gehron Robey P., Laig-Webster M., Chiong W. (1997). PHOG, a candidate gene for involvement in the short stature of Turner syndrome. Hum. Mol. Genet..

[94] Rao E., Weiss B., Fukami M., Rump A., Niesler B., Mertz A., Muroya K., Binder G., Kirsch S., Winkelmann M. (1997). Pseudoautosomal deletions encompassing a novel homeobox gene cause growth failure in idiopathic short stature and Turner syndrome. Nat. Genet..

[95] Lencz T., Morgan T.V., Athanasiou M., Dain B., Reed C.R., Kane J.M., Kucherlapati R., Malhotra A.K. (2007). Converging evidence for a pseudoautosomal cytokine receptor gene locus in schizophrenia. Mol. Psychiatr..

[96] Flaquer A., Jamra R.A., Etterer K., Díaz G.O., Rivas F., Rietschel M., Cichon S., Nöthen M.M., Strauch K. (2010). A new susceptibility locus for bipolar affective disorder in PAR1 on Xp22.3/Yp11.3. Am. J. Med. Genet. B. Neuropsychiatr. Genet..

[97] Helena Mangs A., Morris B.J. (2007). The Human Pseudoautosomal Region (PAR): Origin, Function and Future. Curr. Genom..

[98] Gabriel-Robez O., Rumpler Y., Ratomponirina C., Petit C., Levilliers J., Croquette M.F., Couturier J. (1990). Deletion of the pseudoautosomal region and lack of sex-chromosome pairing at pachytene in two infertile men carrying an X;Y translocation. Cytogenet. Cell Genet..

[99] Mohandas T.K., Speed R.M., Passage M.B., Yen P.H., Chandley A.C., Shapiro L.J. (1992). Role of the pseudoautosomal region in sex-chromosome pairing during male meiosis: Meiotic studies in a man with a deletion of distal Xp. Am. J. Hum. Genet..

[100] Hassold T.J., Sherman S.L., Pettay D., Page D.C., Jacobs P.A. (1991). XY chromosome nondisjunction in man is associated with diminished recombination in the pseudoautosomal region. Am. J. Hum. Genet..

[101] Shi Q., Martin R.H. (2001). Aneuploidy in human spermatozoa: FISH analysis in men with constitutional chromosomal abnormalities, and in infertile men. Reproduction.

[102] Kauppi L., Jasin M., Keeney S. (2012). The tricky path to recombining X and Y chromosomes in meiosis. Ann. N. Y. Acad. Sci..

[103] Veerappa A.M., Padakannaya P., Ramachandra N.B. (2013). Copy number variation-based polymorphism in a new pseudoautosomal region 3 (PAR3) of a human X-chromosome-transposed region (XTR) in the Y chromosome. Funct. Integr. Genom..

[104] Colaco S., Modi D. (2018). Genetics of the human Y chromosome and its association with male infertility. Reprod. Biol. Endocrinol..

[105] Jorgez C.J., Weedin J.W., Sahin A., Tannour-Louet M., Han S., Bournat J.C., Mielnik A., Cheung S.W., Nangia A.K., Schlegel P.N. (2011). Aberrations in pseudoautosomal regions (PARs) found in infertile men with Y-chromosome microdeletions. J. Clin. Endocrinol. Metab..

[106] Chianese C., Lo Giacco D., Tüttelmann F., Ferlin A., Ntostis P., Vinci S., Balercia G., Ars E., Ruiz-Castañé E., Giglio S. (2013). Y-chromosome microdeletions are not associated with SHOX haploinsufficiency. Hum. Reprod..

[107] Castro A., Rodríguez F., Flórez M., López P., Curotto B., Martínez D., Maturana A., Lardone M.C., Palma C., Mericq V. (2017). Pseudoautosomal abnormalities in terminal AZFb+c deletions are associated with isochromosomes Yp and may lead to abnormal growth and neuropsychiatric function. Hum. Reprod..

[108] Sinclair A.H., Berta P., Palmer M.S., Hawkins J.R., Griffiths B.L., Smith M.J., Foster J.W., Frischauf A.M., Lovell-Badge R., Goodfellow P.N. (1990). A gene from the human sex-determining region encodes a protein with homology to a conserved DNA-binding motif. Nature.

[109] She Z.-Y., Yang W.-X. (2017). Sry and SoxE genes: How they participate in mammalian sex determination and gonadal development?. Semin. Cell Dev. Biol..

[110] Kashimada K., Koopman P. (2010). Sry: the master switch in mammalian sex determination. Development.

[111] Vogt P.H., Besikoglu B., Bettendorf M., Frank-Herrmann P., Zimmer J., Bender U., Knauer-Fischer S., Choukair D., Sinn P., Lau Y.-F.C. (2019). Gonadoblastoma Y locus genes expressed in germ cells of individuals with dysgenetic gonads and a Y chromosome in their karyotypes include DDX3Y and TSPY. Hum. Reprod..

[112] Hersmus R., van Bever Y., Wolffenbuttel K.P., Biermann K., Cools M., Looijenga L.H.J. (2017). The biology of germ cell tumors in disorders of sex development. Clin. Genet..

[113] Kido T., Lau Y.-F.C. (2015). Roles of the Y chromosome genes in human cancers. Asian J. Androl..

[114] Anık A., Çatlı G., Abacı A., Böber E. (2013). 46,XX male disorder of sexual development:a case report. J. Clin. Res. Pediatr. Endocrinol..

[115] Bashamboo A., Rahman M.M., Prasad A., Chandy S.P., Ahmad J., Ali S. (2005). Fate of SRY, PABY, DYS1, DYZ3 and DYZ1 loci in Indian patients harbouring sex chromosomal anomalies. Mol. Hum. Reprod..

[116] Dehghani M., Rossi E., Vetro A., Russo G., Hashemian Z., Zuffardi O. (2014). A newborn with ambiguous genitalia and a complex X;Y rearrangement. Iran. J. Reprod. Med..

[117] Premi S., Srivastava J., Chandy S.P., Ahmad J., Ali S. (2006). Tandem duplication and copy number polymorphism of the SRY gene in patients with sex chromosome anomalies and males exposed to natural background radiation. MHR Basic Sci. Reprod. Med..

[118] Kumari A., Yadav S.K., Misro M.M., Ahmad J., Ali S. (2015). Copy number variation and microdeletions of the Y chromosome linked genes and loci across different categories of Indian infertile males. Sci. Rep..

[119] Jangravi Z., Alikhani M., Arefnezhad B., Sharifi Tabar M., Taleahmad S., Karamzadeh R., Jadaliha M., Mousavi S.A., Ahmadi Rastegar D., Parsamatin P. (2013). A fresh look at the male-specific region of the human Y chromosome. J. Proteome Res..

[120] Massaia A., Xue Y. (2017). Human Y chromosome copy number variation in the next generation sequencing era and beyond. Hum. Genet..

[121] Johansson M.M., Van Geystelen A., Larmuseau M.H.D., Djurovic S., Andreassen O.A., Agartz I., Jazin E. (2015). Microarray Analysis of Copy Number Variants on the Human Y Chromosome Reveals Novel and Frequent Duplications Overrepresented in Specific Haplogroups. PLoS ONE.

[122] Chen J.-M., Cooper D.N., Chuzhanova N., Férec C., Patrinos G.P. (2007). Gene conversion: Mechanisms, evolution and human disease. Nat. Rev. Genet..

[123] Rosser Z.H., Balaresque P., Jobling M.A. (2009). Gene Conversion between the X Chromosome and the Male-Specific Region of the Y Chromosome at a Translocation Hotspot. Am. J. Hum. Genet..

[124] Trombetta B., Sellitto D., Scozzari R., Cruciani F. (2014). Inter- and intraspecies phylogenetic analyses reveal extensive X-Y gene conversion in the evolution of gametologous sequences of human sex chromosomes. Mol. Biol. Evol..

[125] Trombetta B., D’Atanasio E., Cruciani F. (2017). Patterns of Inter-Chromosomal Gene Conversion on the Male-Specific Region of the Human Y Chromosome. Front. Genet..

[126] Trombetta B., Cruciani F. (2017). Y chromosome palindromes and gene conversion. Hum. Genet..

[127] Trombetta B., Fantini G., D’Atanasio E., Sellitto D., Cruciani F. (2016). Evidence of extensive non-allelic gene conversion among LTR elements in the human genome. Sci. Rep..

[128] Skaletsky H., Kuroda-Kawaguchi T., Minx P.J., Cordum H.S., Hillier L., Brown L.G., Repping S., Pyntikova T., Ali J., Bieri T. (2003). The male-specific region of the human Y chromosome is a mosaic of discrete sequence classes. Nature.

[129] Schwartz A., Chan D.C., Brown L.G., Alagappan R., Pettay D., Disteche C., McGillivray B., de la Chapelle A., Page D.C. (1998). Reconstructing hominid Y evolution: X-homologous block, created by X-Y transposition, was disrupted by Yp inversion through LINE-LINE recombination. Hum. Mol. Genet..

[130] Bukulmez O. (2012). Genetic Aspects of Male Infertility. Male Infertility.

[131] Rozen S., Marszalek J.D., Alagappan R.K., Skaletsky H., Page D.C. (2009). Remarkably Little Variation in Proteins Encoded by the Y Chromosome’s Single-Copy Genes, Implying Effective Purifying Selection. Am. J. Hum. Genet..

[132] Jain M., Kalsi A.K., Kumar P., Halder A., Kumar A., Sharma M. (2017). The Human Y Chromosome. Basics of Human Andrology: A Textbook.

[133] Jobling M.A., Lo I.C.C., Turner D.J., Bowden G.R., Lee A.C., Xue Y., Carvalho-Silva D., Hurles M.E., Adams S.M., Chang Y.M. (2007). Structural variation on the short arm of the human Y chromosome: Recurrent multigene deletions encompassing Amelogenin, Y. Hum. Mol. Genet..

[134] Repping S., van Daalen S.K.M., Brown L.G., Korver C.M., Lange J., Marszalek J.D., Pyntikova T., van der Veen F., Skaletsky H., Page D.C. (2006). High mutation rates have driven extensive structural polymorphism among human Y chromosomes. Nat. Genet..

[135] Skov L., Schierup M.H., Schierup M.H. (2017). Analysis of 62 hybrid assembled human Y chromosomes exposes rapid structural changes and high rates of gene conversion. PLoS Genet..

[136] Teitz L.S., Pyntikova T., Skaletsky H., Page D.C. (2018). Selection Has Countered High Mutability to Preserve the Ancestral Copy Number of Y Chromosome Amplicons in Diverse Human Lineages. Am. J. Hum. Genet..

[137] Bhowmick B.K., Satta Y., Takahata N. (2007). The origin and evolution of human ampliconic gene families and ampliconic structure. Genome Res..

[138] Ye D., Zaidi A.A., Tomaszkiewicz M., Anthony K., Liebowitz C., DeGiorgio M., Shriver M.D., Makova K.D. (2018). High Levels of Copy Number Variation of Ampliconic Genes across Major Human Y Haplogroups. Genome Biol. Evol..

[139] Decarpentrie F., Vernet N., Mahadevaiah S.K., Longepied G., Streichemberger E., Aknin-Seifer I., Ojarikre O.A., Burgoyne P.S., Metzler-Guillemain C., Mitchell M.J. (2012). Human and mouse ZFY genes produce a conserved testis-specific transcript encoding a zinc finger protein with a short acidic domain and modified transactivation potential. Hum. Mol. Genet..

[140] Nakasuji T., Ogonuki N., Chiba T., Kato T., Shiozawa K., Yamatoya K., Tanaka H., Kondo T., Miyado K., Miyasaka N. (2017). Complementary Critical Functions of Zfy1 and Zfy2 in Mouse Spermatogenesis and Reproduction. PLoS Genet..

[141] Müller U., Kirkels V.G., Scheres J.M. (1992). Absence of Turner stigmata in a 46,XYp-female. Hum. Genet..

[142] Li Y., Zhang D.J., Qiu Y., Kido T., Lau Y.-F.C. (2017). The Y-located proto-oncogene TSPY exacerbates and its X-homologue TSPX inhibits transactivation functions of androgen receptor and its constitutively active variants. Hum. Mol. Genet..

[143] Krausz C., Giachini C., Forti G. (2010). TSPY and Male Fertility. Genes.

[144] Giachini C., Nuti F., Turner D.J., Laface I., Xue Y., Daguin F., Forti G., Tyler-Smith C., Krausz C. (2009). TSPY1 Copy Number Variation Influences Spermatogenesis and Shows Differences among Y Lineages. J. Clin. Endocrinol. Metab..

[145] Yang X., Leng X., Tu W., Liu Y., Xu J., Pei X., Ma Y., Yang D., Yang Y. (2018). Spermatogenic phenotype of testis-specific protein, Y-encoded, 1 (TSPY1) dosage deficiency is independent of variations in TSPY-like 1 (TSPYL1) and TSPY-like 5 (TSPYL5): A case-control study in a Han Chinese population. Reprod. Fertil. Dev..

[146] Vogt P.H., Edelmann A., Kirsch S., Henegariu O., Hirschmann P., Kiesewetter F., Köhn F.M., Schill W.B., Farah S., Ramos C. (1996). Human Y chromosome azoospermia factors (AZF) mapped to different subregions in Yq11. Hum. Mol. Genet..

[147] Kent-First M., Muallem A., Shultz J., Pryor J., Roberts K., Nolten W., Meisner L., Chandley A., Gouchy G., Jorgensen L. (1999). Defining regions of the Y-chromosome responsible for male infertility and identification of a fourth AZF region (AZFd) by Y-chromosome microdeletion detection. Mol. Reprod. Dev..

[148] Krausz C., Hoefsloot L., Simoni M., Tüttelmann F., European Academy of Andrology, European Molecular Genetics Quality Network (2014). EAA/EMQN best practice guidelines for molecular diagnosis of Y-chromosomal microdeletions: State-of-the-art 2013. Andrology.

[149] Colaco S., Modi D. (2019). Consequences of Y chromosome microdeletions beyond male infertility. J. Assist. Reprod. Genet..

[150] Maan A.A., Eales J., Akbarov A., Rowland J., Xu X., Jobling M.A., Charchar F.J., Tomaszewski M. (2017). The Y chromosome: A blueprint for men’s health?. Eur. J. Hum. Genet..

[151] Yu X.-W., Wei Z.-T., Jiang Y.-T., Zhang S.-L. (2015). Y chromosome azoospermia factor region microdeletions and transmission characteristics in azoospermic and severe oligozoospermic patients. Int. J. Clin. Exp. Med..

[152] Rozen S.G., Marszalek J.D., Irenze K., Skaletsky H., Brown L.G., Oates R.D., Silber S.J., Ardlie K., Page D.C. (2012). AZFc Deletions and Spermatogenic Failure: A Population-Based Survey of 20,000 Y Chromosomes. Am. J. Hum. Genet..

[153] Repping S., Skaletsky H., Brown L., van Daalen S.K.M., Korver C.M., Pyntikova T., Kuroda-Kawaguchi T., de Vries J.W.A., Oates R.D., Silber S. (2003). Polymorphism for a 1.6-Mb deletion of the human Y chromosome persists through balance between recurrent mutation and haploid selection. Nat. Genet..

[154] Cram D.S., Osborne E., McLachlan R.I. (2006). Y chromosome microdeletions: Implications for assisted conception. Med. J. Aust..

[155] Sun C., Skaletsky H., Rozen S., Gromoll J., Nieschlag E., Oates R., Page D.C. (2000). Deletion of azoospermia factor a (AZFa) region of human Y chromosome caused by recombination between HERV15 proviruses. Hum. Mol. Genet..

[156] Kuroda-Kawaguchi T., Skaletsky H., Brown L.G., Minx P.J., Cordum H.S., Waterston R.H., Wilson R.K., Silber S., Oates R., Rozen S. (2001). The AZFc region of the Y chromosome features massive palindromes and uniform recurrent deletions in infertile men. Nat. Genet..

[157] Repping S., Skaletsky H., Lange J., Silber S., van der Veen F., Oates R.D., Page D.C., Rozen S. (2002). Recombination between Palindromes P5 and P1 on the Human Y Chromosome Causes Massive Deletions and Spermatogenic Failure. Am. J. Hum. Genet..

[158] Sun C., Skaletsky H., Birren B., Devon K., Tang Z., Silber S., Oates R., Page D.C. (1999). An azoospermic man with a de novo point mutation in the Y-chromosomal gene USP9Y. Nat. Genet..

[159] Ginalski K., Rychlewski L., Baker D., Grishin N. (2004). V Protein structure prediction for the male-specific region of the human Y chromosome. Proc. Natl. Acad. Sci. USA.

[160] Sargent C.A., Boucher C.A., Kirsch S., Brown G., Weiss B., Trundley A., Burgoyne P., Saut N., Durand C., Levy N. (1999). The critical region of overlap defining the AZFa male infertility interval of proximal Yq contains three transcribed sequences. J. Med. Genet..

[161] Lee K.H., Song G.J., Kang I.S., Kim S.W., Paick J.-S., Chung C.H., Rhee K. (2003). Ubiquitin-specific protease activity of USP9Y, a male infertility gene on the Y chromosome. Reprod. Fertil. Dev..

[162] Luddi A., Margollicci M., Gambera L., Serafini F., Cioni M., De Leo V., Balestri P., Piomboni P. (2009). Spermatogenesis in a Man with Complete Deletion of *USP9Y*. N. Engl. J. Med..

[163] Foresta C., Ferlin A., Moro E. (2000). Deletion and expression analysis of AZFa genes on the human Y chromosome revealed a major role for DBY in male infertility. Hum. Mol. Genet..

[164] Ditton H.J., Zimmer J., Kamp C., Rajpert-De Meyts E., Vogt P.H. (2004). The AZFa gene DBY (DDX3Y) is widely transcribed but the protein is limited to the male germ cells by translation control. Hum. Mol. Genet..

[165] Nailwal M., Chauhan J.B. (2017). Computational Analysis of High Risk Missense Variant in Human UTY Gene: A Candidate Gene of AZFa Sub-region. J. Reprod. Infertil..

[166] Lee M.G., Norman J., Shilatifard A., Shiekhattar R. (2007). Physical and functional association of a trimethyl H3K4 demethylase and Ring6a/MBLR, a polycomb-like protein. Cell.

[167] Akimoto C., Kitagawa H., Matsumoto T., Kato S. (2008). Spermatogenesis-specific association of SMCY and MSH5. Genes Cells.

[168] Fan Y., Silber S.J. (1993). Y Chromosome Infertility.

[169] Lopes A.M., Miguel R.N., Sargent C.A., Ellis P.J., Amorim A., Affara N.A. (2010). The human RPS4 paralogue on Yq11.223 encodes a structurally conserved ribosomal protein and is preferentially expressed during spermatogenesis. BMC Mol. Biol..

[170] Andrés O., Kellermann T., López-Giráldez F., Rozas J., Domingo-Roura X., Bosch M. (2008). RPS4Y gene family evolution in primates. BMC Evol. Biol..

[171] Lahn B.T., Page D.C. (2000). A human sex-chromosomal gene family expressed in male germ cells and encoding variably charged proteins. Hum. Mol. Genet..

[172] Navarro-Costa P., Plancha C.E., Gonçalves J. (2010). Genetic dissection of the AZF regions of the human Y chromosome: Thriller or filler for male (in)fertility?. J. Biomed. Biotechnol..

[173] Kichine E., Rozé V., Di Cristofaro J., Taulier D., Navarro A., Streichemberger E., Decarpentrie F., Metzler-Guillemain C., Lévy N., Chiaroni J. (2012). HSFY genes and the P4 palindrome in the AZFb interval of the human Y chromosome are not required for spermatocyte maturation. Hum. Reprod..

[174] Elliott D.J., Millar M.R., Oghene K., Ross A., Kiesewetter F., Pryor J., McIntyre M., Hargreave T.B., Saunders P.T., Vogt P.H. (1997). Expression of RBM in the nuclei of human germ cells is dependent on a critical region of the Y chromosome long arm. Proc. Natl. Acad. Sci. USA.

[175] Tsuei D.-J., Hsu H.-C., Lee P.-H., Jeng Y.-M., Pu Y.-S., Chen C.-N., Lee Y.-C., Chou W.-C., Chang C.-J., Ni Y.-H. (2004). RBMY, a male germ cell-specific RNA-binding protein, activated in human liver cancers and transforms rodent fibroblasts. Oncogene.

[176] Elliott D.J. (2000). RBMY genes and AZFb deletions. J. Endocrinol. Invest..

[177] Venables J.P., Elliott D.J., Makarova O.V., Makarov E.M., Cooke H.J., Eperon I.C. (2000). RBMY, a probable human spermatogenesis factor, and other hnRNP G proteins interact with Tra2beta and affect splicing. Hum. Mol. Genet..

[178] Lingenfelter P.A., Delbridge M.L., Thomas S., Hoekstra H.E., Mitchell M.J., Graves J.A., Disteche C.M. (2001). Expression and conservation of processed copies of the RBMX gene. Mamm. Genome.

[179] Chai N.N., Salido E.C., Yen P.H. (1997). Multiple functional copies of the RBM gene family, a spermatogenesis candidate on the human Y chromosome. Genomics.

[180] Yan Y., Yang X., Liu Y., Shen Y., Tu W., Dong Q., Yang D., Ma Y., Yang Y. (2017). Copy number variation of functional RBMY1 is associated with sperm motility: An azoospermia factor-linked candidate for asthenozoospermia. Hum. Reprod..

[181] Tessari A., Salata E., Ferlin A., Bartoloni L., Slongo M.L., Foresta C. (2004). Characterization of HSFY, a novel AZFb gene on the Y chromosome with a possible role in human spermatogenesis. Mol. Hum. Reprod..

[182] Shinka T., Sato Y., Chen G., Naroda T., Kinoshita K., Unemi Y., Tsuji K., Toida K., Iwamoto T., Nakahori Y. (2004). Molecular characterization of heat shock-like factor encoded on the human Y chromosome, and implications for male infertility. Biol. Reprod..

[183] Vinci G., Raicu F., Popa L., Popa O., Cocos R., McElreavey K. (2005). A deletion of a novel heat shock gene on the Y chromosome associated with azoospermia. Mol. Hum. Reprod..

[184] Sato Y., Yoshida K., Shinka T., Nozawa S., Nakahori Y., Iwamoto T. (2006). Altered expression pattern of heat shock transcription factor, Y chromosome (HSFY) may be related to altered differentiation of spermatogenic cells in testes with deteriorated spermatogenesis. Fertil. Steril..

[185] Kinoshita K., Shinka T., Sato Y., Kurahashi H., Kowa H., Chen G., Umeno M., Toida K., Kiyokage E., Nakano T. (2006). Expression analysis of a mouse orthologue of HSFY, a candidate for the azoospermic factor on the human Y chromosome. J. Med. Invest..

[186] Halder A., Kumar P., Jain M., Iyer V.K. (2017). Copy number variations in testicular maturation arrest. Andrology.

[187] Tahmasbpour E., Balasubramanian D., Agarwal A. (2014). A multi-faceted approach to understanding male infertility: Gene mutations, molecular defects and assisted reproductive techniques (ART). J. Assist. Reprod. Genet..

[188] Stouffs K., Lissens W., Van Landuyt L., Tournaye H., Van Steirteghem A., Liebaers I. (2001). Characterization of the genomic organization, localization and expression of four PRY genes (PRY1, PRY2, PRY3 and PRY4). Mol. Hum. Reprod..

[189] Stouffs K., Lissens W., Verheyen G., Van Landuyt L., Goossens A., Tournaye H., Van Steirteghem A., Liebaers I. (2004). Expression pattern of the Y-linked PRY gene suggests a function in apoptosis but not in spermatogenesis. Mol. Hum. Reprod..

[190] Vogt P.H. (1998). Human chromosome deletions in Yq11, AZF candidate genes and male infertility: History and update. Mol. Hum. Reprod..

[191] Ferlin A., Raicu F., Gatta V., Zuccarello D., Palka G., Foresta C. (2007). Male infertility: Role of genetic background. Reprod. Biomed. Online.

[192] O’Flynn O’Brien K.L., Varghese A.C., Agarwal A. (2010). The genetic causes of male factor infertility: A review. Fertil. Steril..

[193] Lahn B.T., Tang Z.L., Zhou J., Barndt R.J., Parvinen M., Allis C.D., Page D.C. (2002). Previously uncharacterized histone acetyltransferases implicated in mammalian spermatogenesis. Proc. Natl. Acad. Sci. USA.

[194] Vogt P.H. (2005). Azoospermia factor (AZF) in Yq11: Towards a molecular understanding of its function for human male fertility and spermatogenesis. Reprod. Biomed. Online.

[195] Machev N., Saut N., Longepied G., Terriou P., Navarro A., Levy N., Guichaoua M., Metzler-Guillemain C., Collignon P., Frances A.-M. (2004). Sequence family variant loss from the AZFc interval of the human Y chromosome, but not gene copy loss, is strongly associated with male infertility. J. Med. Genet..

[196] Ghorbel M., Baklouti-Gargouri S., Keskes R., Chakroun N., Sellami A., Fakhfakh F., Ammar-Keskes L. (2014). Deletion of CDY1b copy of Y chromosome CDY1 gene is a risk factor of male infertility in Tunisian men. Gene.

[197] Zou S.W., Zhang J.C., Zhang X.D., Miao S.Y., Zong S.D., Sheng Q., Wang L.F. (2003). Expression and localization of VCX/Y proteins and their possible involvement in regulation of ribosome assembly during spermatogenesis. Cell Res..

[198] Lahn B.T., Page D.C. (1997). Functional coherence of the human Y chromosome. Science.

[199] Wong E.Y., Tse J.Y., Yao K.-M., Tam P.-C., Yeung W.S. (2002). VCY2 protein interacts with the HECT domain of ubiquitin-protein ligase E3A. Biochem. Biophys. Res. Commun..

[200] Lu C., Jiang J., Zhang R., Wang Y., Xu M., Qin Y., Lin Y., Guo X., Ni B., Zhao Y. (2014). Gene copy number alterations in the azoospermia-associated AZFc region and their effect on spermatogenic impairment. MHR Basic Sci. Reprod. Med..

[201] Tse J.Y.M., Wong E.Y.M., Cheung A.N.Y., O W.S., Tam P.C., Yeung W.S.B. (2003). Specific expression of VCY2 in human male germ cells and its involvement in the pathogenesis of male infertility. Biol. Reprod..

[202] Wong E.Y.M., Tse J.Y.M., Yao K.-M., Lui V.C.H., Tam P.-C., Yeung W.S.B. (2004). Identification and Characterization of Human VCY2-Interacting Protein: VCY2IP-1, a Microtubule-Associated Protein-Like Protein. Biol. Reprod..

[203] Hughes J.F., Skaletsky H., Page D.C. (2012). Sequencing of rhesus macaque Y chromosome clarifies origins and evolution of the DAZ (Deleted in AZoospermia) genes. Bioessays.

[204] Saxena R., Brown L.G., Hawkins T., Alagappan R.K., Skaletsky H., Reeve M.P., Reijo R., Rozen S., Dinulos M.B., Disteche C.M. (1996). The DAZ gene cluster on the human Y chromosome arose from an autosomal gene that was transposed, repeatedly amplified and pruned. Nat. Genet..

[205] Fu X.-F., Cheng S.-F., Wang L.-Q., Yin S., De Felici M., Shen W. (2015). DAZ Family Proteins, Key Players for Germ Cell Development. Int. J. Biol. Sci..

[206] Moro E., Ferlin A., Yen P.H., Franchi P.G., Palka G., Foresta C. (2000). Male infertility caused by a de novo partial deletion of the DAZ cluster on the Y chromosome. J. Clin. Endocrinol. Metab..

[207] Menke D.B., Mutter G.L., Page D.C. (1997). Expression of DAZ, an azoospermia factor candidate, in human spermatogonia. Am. J. Hum. Genet..

[208] Kee K., Angeles V.T., Flores M., Nguyen H.N., Reijo Pera R.A. (2009). Human DAZL, DAZ and BOULE genes modulate primordial germ-cell and haploid gamete formation. Nature.

[209] Habermann B., Mi H.F., Edelmann A., Bohring C., Bäckert I.T., Kiesewetter F., Aumüller G., Vogt P.H. (1998). DAZ (Deleted in AZoospermia) genes encode proteins located in human late spermatids and in sperm tails. Hum. Reprod..

[210] Saxena R., de Vries J.W., Repping S., Alagappan R.K., Skaletsky H., Brown L.G., Ma P., Chen E., Hoovers J.M., Page D.C. (2000). Four DAZ genes in two clusters found in the AZFc region of the human Y chromosome. Genomics.

[211] Reijo R., Lee T.-Y., Salo P., Alagappan R., Brown L.G., Rosenberg M., Rozen S., Jaffe T., Straus D., Hovatta O. (1995). Diverse spermatogenic defects in humans caused by Y chromosome deletions encompassing a novel RNA–binding protein gene. Nat. Genet..

[212] Yang Y., Xiao C.-Y., A Z.-C., Zhang S.-Z., Li X., Zhang S.-X. (2006). DAZ1/DAZ2 cluster deletion mediated by gr/gr recombination per se may not be sufficient for spermatogenesis impairment: A study of Chinese normozoospermic men. Asian J. Androl..

[213] Lin Y.-W., Hsu L.C.-L., Kuo P.-L., Huang W.J., Chiang H.-S., Yeh S.-D., Hsu T.-Y., Yu Y.-H., Hsiao K.-N., Cantor R.M. (2007). Partial duplication at AZFc on the Y chromosome is a risk factor for impaired spermatogenesis in Han Chinese in Taiwan. Hum. Mutat..

[214] Noordam M.J., Westerveld G.H., Hovingh S.E., van Daalen S.K.M., Korver C.M., van der Veen F., van Pelt A.M.M., Repping S. (2011). Gene copy number reduction in the azoospermia factor c (AZFc) region and its effect on total motile sperm count. Hum. Mol. Genet..

[215] Ye J., Ma L., Yang L., Wang J., Wang Y., Guo H., Gong N., Nie W., Zhao S. (2013). Partial AZFc duplications not deletions are associated with male infertility in the Yi population of Yunnan Province, China. J. Zhejiang Univ. Sci. B.

[216] Lu C., Wang Y., Zhang F., Lu F., Xu M., Qin Y., Wu W., Li S., Song L., Yang S. (2013). DAZ duplications confer the predisposition of Y chromosome haplogroup K* to non-obstructive azoospermia in Han Chinese populations. Hum. Reprod..

[217] Manz E., Alkan M., Bühler E., Schmidtke J. (1992). Arrangement of DYZ1 and DYZ2 repeats on the human Y-chromosome: A case with presence of DYZ1 and absence of DYZ2. Mol. Cell. Probes.

[218] Cooke H. (1976). Repeated sequence specific to human males. Nature.

[219] Unnérus V., Fellman J., De la Chapelle A. (1967). The length of the human Y chromosome. Cytogenetics.

[220] Akkari Y., Lawce H., Kelson S., Smith C., Davis C., Boyd L., Magenis R.E., Olson S. (2005). Y chromosome heterochromatin of differing lengths in two cell populations of the same individual. Prenat. Diagn..

[221] Cotter P.D., Norton M.E. (2005). Y chromosome heterochromatin variation detected at prenatal diagnosis. Prenat. Diagn..

[222] Bobrow M., Pearson P.L., Pike M.C., El-Alfi O.S. (1971). Length variation in the quinacrine-binding segment of human Y chromosomes of different sizes. Cytogenetics.

[223] Laberge C., Gagne R. (1971). Quinacrine mustard staining solves the length variations of the human Y chromosome. Johns Hopkins Med. J..

[224] Lau Y.F., Schonberg S. (1984). A male-specific DNA probe detects heterochromatin sequences in a familial Yq- chromosome. Am. J. Hum. Genet..

[225] Schmid M., Guttenbach M., Nanda I., Studer R., Epplen J.T. (1990). Organization of DYZ2 repetitive DNA on the human Y chromosome. Genomics.

[226] Cooke H.J., Noel B. (1979). Confirmation of Y/autosome translocation using recombinant DNA. Hum. Genet..

[227] Yan J., Fan L., Zhao Y., You L., Wang L., Zhao H., Li Y., Chen Z.-J. (2011). DYZ1 copy number variation, Y chromosome polymorphism and early recurrent spontaneous abortion/early embryo growth arrest. Eur. J. Obstet. Gynecol. Reprod. Biol..

[228] Malaspina P., Persichetti F., Novelletto A., Iodice C., Terrenato L., Wolfe J., Ferraro M., Prantera G. (1990). The human Y chromosome shows a low level of DNA polymorphism. Ann. Hum. Genet..

[229] Babcock M., Yatsenko S., Stankiewicz P., Lupski J.R., Morrow B.E. (2007). AT-rich repeats associated with chromosome 22q11.2 rearrangement disorders shape human genome architecture on Yq12. Genome Res..

[230] Mathias N. (2013). Y chromosome DNA polymorphisms and human evolution. Ph.D. Thesis.

[231] Jehan Z., Vallinayagam S., Tiwari S., Pradhan S., Singh L., Suresh A., Reddy H.M., Ahuja Y.R., Jesudasan R.A. (2007). Novel noncoding RNA from human Y distal heterochromatic block (Yq12) generates testis-specific chimeric CDC2L2. Genome Res..

[232] Garcia-Blanco M.A. (2003). Messenger RNA reprogramming by spliceosome-mediated RNA trans-splicing. J. Clin. Invest..

[233] Nazarenko S.A., Puzyrev V.P., Protasov K.T., Ostrovskaia M.G. (1989). Heterochromatin of the Y-chromosome and variability of human morphophysiological traits. Genetika.

[234] Yakin K., Balaban B., Urman B. (2005). Is there a possible correlation between chromosomal variants and spermatogenesis?. Int. J. Urol..

[235] Navarro-Costa P., Plancha C.E. (2011). Heterochromatin: the hidden epigenetic geography of the Y chromosome. Hum. Reprod. Update.

[236] Lemos B., Branco A.T., Hartl D.L. (2010). Epigenetic effects of polymorphic Y chromosomes modulate chromatin components, immune response, and sexual conflict. Proc. Natl. Acad. Sci. USA.

[237] Zhang F., Gu W., Hurles M.E., Lupski J.R. (2009). Copy Number Variation in Human Health, Disease, and Evolution. Annu. Rev. Genomics Hum. Genet..

[238] Lopes A.M., Aston K.I., Thompson E., Carvalho F., Gonçalves J., Huang N., Matthiesen R., Noordam M.J., Quintela I., Ramu A. (2013). Human spermatogenic failure purges deleterious mutation load from the autosomes and both sex chromosomes, including the gene DMRT1. PLoS Genet..

[239] Huang N., Wen Y., Guo X., Li Z., Dai J., Ni B., Yu J., Lin Y., Zhou W., Yao B. (2015). A Screen for Genomic Disorders of Infertility Identifies MAST2 Duplications Associated with Nonobstructive Azoospermia in Humans. Biol. Reprod..

[240] Eggers S., DeBoer K.D., van den Bergen J., Gordon L., White S.J., Jamsai D., McLachlan R.I., Sinclair A.H., O’Bryan M.K. (2015). Copy number variation associated with meiotic arrest in idiopathic male infertility. Fertil. Steril..

[241] Ji J., Qin Y., Wang R., Huang Z., Zhang Y., Zhou R., Song L., Ling X., Hu Z., Miao D. (2016). Copy number gain of VCX, X-linked multi-copy gene, leads to cell proliferation and apoptosis during spermatogenesis. Oncotarget.

[242] Nakamura S., Miyado M., Saito K., Katsumi M., Nakamura A., Kobori Y., Tanaka Y., Ishikawa H., Yoshida A., Okada H. (2017). Next-generation sequencing for patients with non-obstructive azoospermia: implications for significant roles of monogenic/oligogenic mutations. Andrology.

[243] Tüttelmann F., Ruckert C., Röpke A. (2018). Disorders of spermatogenesis. Med. Genet..

[244] Ahmadi Rastegar D., Sharifi Tabar M., Alikhani M., Parsamatin P., Sahraneshin Samani F., Sabbaghian M., Sadighi Gilani M.A., Mohammad Ahadi A., Mohseni Meybodi A., Piryaei A. (2015). Isoform-Level Gene Expression Profiles of Human Y Chromosome Azoospermia Factor Genes and Their X Chromosome Paralogs in the Testicular Tissue of Non-Obstructive Azoospermia Patients. J. Proteome Res..

